# Integrated Phytochemical Profiling and Bioactivity Evaluation of *Micromeria nervosa*, with Emphasis on Antimicrobial and Antiviral Properties

**DOI:** 10.3390/antibiotics15040374

**Published:** 2026-04-06

**Authors:** Ljuboš Ušjak, Krystyna Skalicka-Woźniak, Łukasz Kulinowski, Łukasz Świątek, Violeta Milutinović, Kinga Salwa, Anastazja Boguszewska, Izabela Korona-Glowniak, Katarzyna Suśniak, Marjan Niketić, Jelena Kukić Marković, Silvana Petrović

**Affiliations:** 1 Department of Pharmacognosy, Faculty of Pharmacy, University of Belgrade, Vojvode Stepe 450, 11221 Belgrade, Serbia; violeta.milutinovic@pharmacy.bg.ac.rs (V.M.); jelena.kukic@pharmacy.bg.ac.rs (J.K.M.); 2 Department of Natural Products Chemistry, Medical University of Lublin, 1 Chodzki Street, 20-093 Lublin, Poland; kskalicka@pharmacognosy.org (K.S.-W.); lukasz.kulinowski@umlub.edu.pl (Ł.K.); 3 Department of Virology with Viral Diagnostics Laboratory, Medical University of Lublin, 1 Chodzki Street, 20-093 Lublin, Poland; lukasz.swiatek@umlub.edu.pl (Ł.Ś.); kinga.salwa@umlub.edu.pl (K.S.); anastazja.boguszewska@umlub.edu.pl (A.B.); 4 Department of Pharmaceutical Microbiology, Medical University of Lublin, 1 Chodzki Street, 20-093 Lublin, Poland; izabela.korona-glowniak@umlub.edu.pl (I.K.-G.); katarzyna.susniak@umlub.edu.pl (K.S.); 5 Natural History Museum, Njegoševa 51, 11000 Belgrade, Serbia; 6 Serbian Academy of Sciences and Arts, Kneza Mihaila 35/II, 11000 Belgrade, Serbia

**Keywords:** LC-DAD-QTOF-MS/MS, GC-FID/MS, antibacterial, anticandidal, antiviral, cytotoxic, antioxidant, correlation analysis, molecular docking, molecular dynamics

## Abstract

**Background/Objectives**: Lamiaceae species are valuable sources of bioactive natural products, often associated with anti-infective properties. This study investigated chemical composition and bioactivities of dry hydroethanolic extracts and essential oils from *Micromeria nervosa* (Desf.) Benth. aerial parts from two localities. **Methods**: Extracts and essential oils were analyzed using LC-DAD-QTOF-MS/MS and GC-FID/MS, respectively. Antimicrobial activity was assessed against 14 strains (microdilution method), and antiviral activity against three viruses by determining cytopathic effects, viral titers (end-point dilution assay) and viral loads (qPCR/RT-qPCR). Cytotoxicity was evaluated on three cancer cell lines (MTT assay) and antioxidant potential using three colorimetric tests. Composition–activity correlation was statistically analyzed; in silico molecular docking/dynamics simulations were performed. **Results**: Thirty-five compounds were annotated in extracts, including 30 reported for the first time in this species, with rosmarinic acid as the main component. Essential oils contained 31 constituents, dominated by carvacrol. Newly detected phenolics included lithospermic acid and several salvianolic and clinopodic acids. Extracts and oils exhibited notable antibacterial activity, especially against five Gram-positive strains (MIC = 0.313–2.5 mg/mL), and oils showed marked anticandidal effects (MIC = 0.313–0.625 mg/mL) and enhanced cytotoxicity against colon, gastric and hypopharyngeal cancer cells (selectivity indices ≥ 1.66). Extracts displayed potent antiviral activity against human herpesvirus 1 (HHV-1) and adenovirus Ad5, reducing cytopathic effects and viral titers, with qPCR revealing decreased HHV-1 load. In silico analysis suggested HHV-1 glycoprotein D binding. Extracts also showed strong antioxidant potential. **Conclusions**: These findings demonstrate that *M. nervosa* is a rich source of compounds with antimicrobial/antiviral, cytotoxic and antioxidant activities, warranting further research.

## 1. Introduction

Plants from the mint family (Lamiaceae) serve as important sources of raw materials for the pharmaceutical, cosmetic and food industries, and are widely used as folk medicines and culinary ingredients [1,2,3]. In the context of the increasing emergence of antimicrobial and antiviral resistance, products derived from these plants have gained considerable attention as potential sources of novel antimicrobial and antiviral agents or adjuvants, with demonstrated activity against clinically relevant pathogens [4,5]. In recent years, particularly following the COVID-19 pandemic, the search for novel antiviral agents has intensified, driven by the global burden of viral infections, limitations of current therapies, and the need for safe, effective and accessible alternatives [5]. Furthermore, Lamiaceae species are also used as natural flavoring agents, food preservatives and functional food components, and are commonly consumed as aromatic herbal teas [1,2,3]. These diverse applications stem from their richness in essential oils and/or polyphenols, such as flavonoids and phenolic acids, which exhibit antibacterial, antifungal, antiviral, antioxidant, anti-inflammatory, chemopreventive, and other bioactivities [3]. For example, essential oil components such as thymol and carvacrol exhibit pronounced antibacterial and antifungal activity, whereas polyphenols, particularly phenolic acids, have attracted considerable attention due to their ability to interfere with different stages of viral infection [3,5]. Common medicinal and culinary plants in this family include mint, sage, rosemary, savory, oregano, hyssop, thyme and lavender [3].

The genus *Micromeria* Benth. comprises approximately 70 species distributed in Africa, Asia, Europe and North America, with the vast majority of them growing in the Mediterranean region and Canary Islands [6]. The plants of this genus are known for producing essential oils, which are often rich in oxygenated monoterpenes, such as thymol and carvacrol [7,8]. Beyond essential oils, fewer studies have focused on the more polar and less volatile metabolites of *Micromeria* species, among which rosmarinic acid—a marker compound of the whole Nepetoideae subfamily—is the most commonly identified [1,7,9]. In Mediterranean folk medicine, the flowering aerial parts (herbs) of various plants from this genus are valued for their anti-inflammatory and antiseptic properties. They are usually prepared as teas, which are applied to treat digestive, cardiovascular and respiratory ailments, as well as to promote wound healing. Furthermore, previous studies have shown that *Micromeria* species exhibit a broad spectrum of bioactivities, including antibacterial, antifungal, antioxidant, anticholinesterase, tyrosinase inhibitory and antinociceptive effects, making them valuable candidates for various pharmaceutical and cosmetic formulations and for use as natural food additives and functional food components [1,7,8,9].

*Micromeria nervosa* (Desf.) Benth. is a subshrub, typically 15–40 cm tall, primarily thriving in the subtropical biome of the Mediterranean region [6]. It commonly inhabits arid rocky outcrops and rock crevices. The plant features square-shaped, densely hairy stems and small, hairy leaves (5–15 mm long), typically ovate to lanceolate with an acute tip. The spike-like inflorescence bears narrow, densely pubescent calyces (3–4.5 mm) with elongated teeth, while the corollas (5 mm) are usually pink to purple, with single-lobed upper lips and three-lobed lower lips [10]. It has gained increasing attention from the scientific community, especially in recent years. In the dry ethyl acetate, methanol and aqueous extracts of the aerial parts from Turkey, rosmarinic acid was revealed to be the main phenolic compound. The aqueous extract demonstrated the highest antioxidant potential, while the ethyl acetate and methanol extracts exhibited the strongest inhibitory effects on *α*-amylase and tyrosinase enzymes [9]. Fractionation of the dry acetone extract prepared from the aerial parts collected in Egypt led to the isolation of a new sesquiterpenoid, micromeriol, and a new sterol, nervosane, along with three known triterpenes and sterols. This extract and the isolated compounds displayed cytotoxicity against cancer cells and antioxidant activity [11]. The dry ethanol extract of the leaves from Tunisia and its different fractions showed antioxidant activity, whereas the diethyl ether fraction also exhibited high antibacterial activity against Gram-positive strains and potent antileishmanial activity against *Leishmania* promastigotes. Ursolic acid, isolated using bioactivity-guided fractionation from the diethyl ether fraction, displayed strong efficacy against promastigote and amastigote forms of *Leishmania*, affecting genes involved in the thiol pathway [12]. Essential oil isolated from these leaves was also analyzed; *α*-pinene and *τ*-cadinol were identified as major components. The oil demonstrated antioxidant activity and significant leishmanicidal activity (targeting thiol pathway genes), with *τ*-cadinol showing in silico the strongest interaction with the CYP51 enzyme (which inhibition could lead to antileishmanial effect) [13]. In the essential oil of aerial parts from Egypt, caryophyllene oxide was the dominant [14]. Different organic and aqueous extracts of the aerial parts collected in the Palestine area exhibited antimicrobial activity, with the ethanol extract being the most effective. Carvacrol was isolated from these aerial parts and revealed as the key antimicrobial component [15]. These promising findings support the need for further in-depth studies of the composition and bioactivity of *M. nervosa*. Therefore, the aim of this work was to investigate chemical composition of the dry hydroethanolic extracts and essential oils of *M. nervosa* herb (leaves and flowers), collected from two localities in Greece (sample_1 in 2022 and sample_2 in 2023), as well as to study their bioactivity (antibacterial, antifungal, antiviral, cytotoxic and antioxidant effects) using a battery of in vitro and in silico assays.

## 2. Results and Discussion

### 2.1. Phytochemical Profiles of Dry Hydroethanolic Extracts

Using high-resolution mass spectrometry (HRMS) data acquired on a LC-DAD-QTOF-MS/MS instrument, a total of 35 compounds were detected in the dry hydroethanolic extracts: 12 phenolic acids (**2**, **3**, **7**, **13**, **21**, **24**–**26**, **28**, **29**, **31** and **33**), four other organic acids (**1** and **4**–**6**), 17 flavonoids (**8**–**12**, **14**–**20**, **22**, **23**, **27**, **30** and **32**) and two diterpenes (**34** and **35**) (Table 1; Figures S1 and S2). In the extract of sample_1 (collected in August 2022 near the city of Nafplio, Greece), 34 compounds were revealed (diterpene **35** was not detected), while in the extract of sample_2 (collected in May 2023 on the Methana Peninsula, Greece), 33 compounds were found (phenolic acids **25** and **33** were absent).

Among the phenolic acids, a depside rosmarinic acid (**21**; pseudomolecular ion at *m*/*z* 359.0748) exhibited fragment ions at *m*/*z* corresponding to the mass of the compounds formed upon cleavage of its ester bond—salvianic acid A (also known as danshensu) and caffeic acid (*m*/*z* 197.0434 and 179.0338, respectively). Both these compounds were also detected in the extracts (compounds **2** and **7**) based on their characteristic fragmentation patterns. Identity of rosmarinic acid (**21**) was confirmed using a commercial standard. Additionally, a hexoside of rosmarinic acid (**13**) was revealed based on the neutral loss of 162 Da, corresponding to anhydrohexose [16,17]. Furthermore, lithospermic acid (also known as lithospermic acid A; **24**) and salvianolic acid H (**26**), both trimers formed by the addition of one more caffeic acid unit to rosmarinic acid (pseudomolecular ions at *m*/*z* 537.1018), were detected. Their mass spectra revealed the fragment ions at *m*/*z* 493.1070–493.1112, 359.0695–359.0729, 339.0423–339.0456, 313.0368–313.0652 and 295.0545–295.0574 (with the one at *m*/*z* 295 being the most prominent in both **24** and **26**), corresponding to the neutral losses of CO_2_ unit (44 Da), anhydrosalvianic acid A unit (180 Da), salvianic acid A unit (198 Da), anhydrosalvianic acid A unit together with CO_2_ unit (224 Da), and salvianic acid A unit together with CO_2_ unit (242 Da), respectively. These signals were accompanied by characteristic fragmentation of rosmarinic acid. It should be noted that in lithospermic acid, the additional caffeic acid unit is attached to rosmarinic acid via a dihydrobenzofuran nucleus, resulting in a different UV spectrum compared to salvianolic acid H [16,18,19,20,21]. Previously, they were both identified in the dry methanol extract of *Thymus dacicus* Borbás (Lamiaceae) flowering aerial parts [22], which served as a reference for confirming their identity in this study. Regarding tetramers, three further salvianolic acid isomers (**28**, **29** and **31**), along with clinopodic acid I or its isomer (**25**), were detected (pseudomolecular ions at *m*/*z* 717.1423). These compounds contain one additional salvianic acid A unit compared to salvianolic acid H and lithospermic acid [21,23]. In the case of tetrameric salvianolic acid isomers (three known are marked with letters E, B and L), two salvianic acid A units are terminal, and the neutral losses of one or both of them or their anhydro forms yield signals at *m*/*z* 537.0811–537.0844, 519.0546–519.0853 (the most prominent one), 357.0477–357.0559, 339.0251–339.0456 and/or 321.0177–321.0340 in the mass spectra. Given its UV spectrum similarity to lithospermic acid, salvianolic acid that elutes first (**28**) is putatively salvianolic acid B (also known as lithospermic acid B), which, like lithospermic acid, contains a dihydrobenzofuran nucleus [21]. Clinopodic acid I also contains a dihydrobenzofuran nucleus, but its terminal units consist of one salvianic acid A and one caffeic acid. Therefore, besides the prominent fragment ion resulting from the salvianic acid A loss (*m*/*z* 519.0850), the mass spectrum of this compound also exhibited the fragmentation ion resulting from anhydrocaffeic acid loss (*m*/*z* 555.1069). Additionally, a hexamer, clinopodic acid O or its isomer (**33**; pseudomolecular ion at *m*/*z* 1075.2115), was detected. This compound consists of two lithospermic acid units, with prominent fragment ions in the mass spectrum corresponding to neutral salvianic acid A loss (*m*/*z* 877.1673) and subsequent loss of the remaining part of the lithospermic acid unit (*m*/*z* 519.0889) [23]. The mass spectra of the remaining simple phenolic acid (**3**) and other detected organic acids (**1** and **4**–**6**) were also consistent with previously published data [16,18].

The classes of flavonoids detected in extracts included both *O*-glycosides (**10**–**12**, **14**–**20**, **23** and **27**) and *C*-glycosides (**8** and **9**). Regarding *O*-glycosides, their mass spectra consisted of the signals corresponding to pseudomolecular ions, aglycones (whose structure was confirmed based on characteristic UV spectra), and, in the case of di-*O*-glycosides, the signals that correspond to loss of one sugar unit. Among these compounds, three luteolin *O*-glycosides (**11**, **15** and **20**; aglycone *m*/*z* 285.0381–285.0391 in negative ion mode), three apigenin *O*-glycosides (**14**, **17** and **23**; aglycone *m*/*z* 271.0572–271.0587 in positive ion mode and *m*/*z* 269.0386 in negative ion mode), as well as *O*-glycosides of hydroxy kaempferol (**10**; aglycone *m*/*z* 303.0475 in positive ion mode), quercetin (**12**; aglycone *m*/*z* 301.0237 in negative ion mode), hesperetin (**16**; aglycone *m*/*z* 301.0676 in negative ion mode), hydroxy apigenin (**18**; aglycone *m*/*z* 287.0529 in positive ion mode), methyl luteolin (**19**; aglycone *m*/*z* 301.0696 in positive ion mode) and methyl apigenin (**27**; aglycone *m*/*z* 285.0733 in positive ion mode) were detected. In the case of sugar units, loss of 162 Da in the mass spectrum was tentatively assigned to hexose (**12**, **14**, **18**, **19** and **27**), loss of 146 Da to deoxyhexose (**19** and **27**), loss of 176 Da to hexuronic acid (**10**, **14**, **15**, **20** and **23**) and loss of 308 Da to deoxyhexosylhexose (**11**, **16** and **17**). Aglycones luteolin (**30**) and apigenin (**32**) were also revealed [16,18]. Using commercial standards, luteolin and apigenin, their 7-*O*-rutinosides and 7-*O*-glucuronides (**11**, **15**, **17**, **23**, **30** and **32**), as well as quercetin 3-*O*-glucoside (**12**) were identified. Regarding *C*-glycosides, the presence of two of them, apigenin 6,8-di-*C*-glucoside (**8**; vicenin 2; identity confirmed using commercial standard) and one luteolin *C*-hexoside (**9**; orientin or isoorientin) was revealed based on characteristic fragmentation patterns and UV spectra. Namely, due to the stronger nature of the C-C bond, in the mass spectra of these two compounds, the fragment ions originating from the loss of fragments of sugar units (e.g., neutral losses of 90 and 120 Da) were observed [24,25]. In addition, one gallocatechin isomer (**22**) was detected, and two diterpenes, carnosol (**34**) and dehydrocarnosol (**35**), were tentatively identified based on their fragmentation patterns [18].

Quantitative chromatographic analysis revealed that the sample_1 extract (194.64 mg/g) was richer in phenolics compared to the sample_2 extract (143.72 mg/g). Phenolic acids were the dominant in both extracts (165.42 mg/g of sample_1 extract and 91.58 mg/g of sample_2 extract), followed by flavonoids (29.22 and 52.14 mg/g). The most abundant compound was rosmarinic acid (**21**; 117.95 and 66.49 mg/g), followed by luteolin 7-*O*-glucuronide (**15**; 11.68 and 27.05 mg/g), apigenin 6,8-di-*C*-glucoside (**8**; 6.00 and 7.28 mg/g), lithospermic acid (**24**; 8.47 and 6.48 mg/g) and different salvianolic acids (**26**, **28**, **29** and **31**; 4.00–20.37 and 0.93–9.22 mg/g). Accordingly, the sample_1 extract exhibited somewhat higher colorimetrically determined total phenolic content (179.06 ± 3.02 mg of gallic acid equivalents, GAE/g of dry extract) than the sample_2 extract (156.12 ± 7.84 mg GAE/g).

**Table 1:** Chemical composition of investigated *M. nervosa* dry hydroethanolic extracts.

Rt 1 ^1^ (min)	Rt 2 ^2^ (min)	Compound	UV Data	QTOF-MS/MS Data						Content (mg/g Dry Extract) ^6^	
			λ_max_ (nm)	Molecular Mass	Calculated Mass	Diff. (ppm)	Molecular Formula	Pseudomol. Ion *m*/*z*	Fragment Ions *m*/*z*	Sample_1	Sample_2
-	5.56	Quinic acid (**1**) [16,18] ^3^	n.r. ^5^	192.0634	191.0561	8.91	C_7_H_12_O_6_	191.0554 [M-H]^−^	145.0510, 129.0544, 115.0380, 101.0596	+ ^7^	+
-	10.64	Salvianic acid A (danshensu; **2**) [16,17]	n.r.	198.0528	197.0455	0.24	C_9_H_10_O_5_	197.0455 [M−H]^−^	179.0348, 151.0347, 135.0445, 123.0334, 109.0283	+	+
-	12.15	Protocatechuic acid hexoside (**3**) [16,18]	n.r.	316.0794	315.0722	−7.10	C_13_H_16_O_9_	315.0744 [M−H]^−^	153.0518	+	+
-	13.44	Hydroxybenzoic acid hexoside (**4**) [16,18]	n.r.	300.0845	299.0772	0.47	C_13_H_16_O_8_	299.0771 [M−H]^−^	137.0237	+	+
-	13.91	Hydroxybenzoic acid (**5**) [16,18]	n.r.	138.0317	137.0244	8.82	C_7_H_6_O_3_	137.0232 [M−H]^−^	108.0209	+	+
-	18.32	Tuberonic acid hexoside (**6**) [16,18]	n.r.	388.1733	387.1661	6.84	C_18_H_28_O_9_	387.1634 [M−H]^−^	207.0947, 101.0210	+	+
-	18.71	Caffeic acid (**7**) [16,17]	n.r.	180.0423	179.0350	3.23	C_9_H_8_O_4_	179.0334 [M−H]^−^	135.0435, 117.0311, 107.0479	+	+
15.30	20.16	Apigenin 6,8-di-*C*-glucoside (vicenin 2; **8**) ^4^ [24,25]	271, 336	594.1585	593.1512	5.38	C_27_H_30_O_15_	593.1480 [M−H]^−^	503.0776, 473.0706, 383.0457, 353.0380	6.00 ± 0.59	7.28 ± 1.40
18.49	22.16	Luteolin *C*-hexoside (orientin/isoorientin; **9**) [24,25]	256 (sh), 272, 350	448.1006	447.0933	4.43	C_21_H_20_O_11_	447.0913 [M−H]^−^	357.0613, 339.0509, 327.0513, 297.0399, 285.0389	0.55 ± 0.02	0.41 ± 0.04
19.74	24.15	Hydroxy kaempferol hexuronide (**10**) [18,19,24]	282, 344	478.0747	479.0820	−2.47	C_21_H_18_O_13_	479.0832 [M+H]^+^	303.0475	1.55 ± 0.31	3.65 ± 0.17
21.35	23.79	Luteolin 7-*O*-rutinoside (scolimoside; **11**) ^4^ [16,18]	256, 268, 348	594.1585	593.1512	4.70	C_27_H_30_O_15_	593.1484 [M−H]^−^	285.0381	3.11 ± 0.36	2.27 ± 0.46
21.77	24.04	Quercetin 3-*O*-glucoside (isoquercitrin; **12**) ^4^ [16,18]	256, 264, 298, 354	464.0955	463.0882	0.00	C_21_H_20_O_12_	463.0882 [M−H]^−^	301.0237, 300.0198	0.50 ± 0.13	0.36 ± 0.00
-	24.81	Rosmarinic acid hexoside (**13**) [16,17]	n.r.	522.1373	521.1301	0.89	C_24_H_26_O_13_	521.1296 [M−H]^−^	359.0656, 323.0526, 197.0307, 179.0221, 161.0126, 135.0346	+	+
22.12	25.26	Apigenin hexosyl hexuronide (**14**) [16,18,24]	268, 338	608.1377	609.1450	−2.78	C_27_H_28_O_16_	609.1467 [M+H]^+^	447.0944, 303.0826, 271.0587	0.76 ± 0.10	0.45 ± 0.06
22.67	25.71	Luteolin 7-*O*-glucuronide (**15**) ^4^ [16]	254, 268, 344	462.0798	461.0725	3.79	C_21_H_18_O_12_	461.0708 [M−H]^−^	285.0391	11.68 ± 0.18	27.05 ± 0.26
23.34	25.41	Hesperetin deoxyhexosyl hexoside (**16**) [19]	226, 284	610.1898	609.1825	6.22	C_28_H_34_O_15_	609.1787 [M−H]^−^	301.0676, 286.0432	0.14 ± 0.01	0.29 ± 0.05
23.72	25.46	Apigenin 7-*O*-rutinoside (isorhoifolin; **17**) ^4^ [16,18,19]	268, 338	578.1636	577.1563	2.39	C_27_H_30_O_14_	577.1549 [M−H]^−^	269.0386	0.33 ± 0.11	0.41 ± 0.21
24.21	26.10	Hydroxy apigenin hexoside (**18**) [16,18]	272, 336	448.1006	449.1078	0.08	C_21_H_20_O_11_	449.1078 [M+H]^+^	287.0529	0.49 ± 0.01	1.29 ± 0.10
24.59	26.62	Methyl luteolin deoxyhexosyl hexoside (**19**) [16,18]	252, 270, 348	608.1741	609.1814	−1.49	C_28_H_32_O_15_	609.1823 [M+H]^+^	463.1229, 301.0696, 286.0468	2.48 ± 0.22	3.18 ± 1.15
-	26.71	Luteolin hexuronide (**20**) [16]	n.r.	462.0798	461.0725	3.79	C_21_H_18_O_12_	461.0708 [M−H]^−^	285.0391	+	+
25.63	27.68	Rosmarinic acid (**21**) ^4^ [16,17]	290, 330	360.0845	359.0772	6.78	C_18_H_16_O_8_	359.0748 [M−H]^−^	197.0434, 179.0338, 161.0235, 135.0442, 133.0238, 123.0444	117.95 ± 3.01	66.49 ± 2.34
-	27.81	Gallocatechin isomer (**22**) [18]	n.r.	306.0740	305.0667	−4.32	C_15_H_14_O_7_	305.0680 [M−H]^−^	225.1131	+	+
25.69	27.85	Apigenin 7-*O*-glucuronide (scutellarin A; **23**) ^4^ [16,18]	268, 338	446.0849	447.0922	1.99	C_21_H_18_O_11_	447.0913 [M+H]^+^	271.0572	tr ^8^	tr
26.38	29.46	Lithospermic acid (**24**) [16,18,19,20,21]	230, 256, 290, 308, 336	538.1111	537.1038	3.81	C_27_H_22_O_12_	537.1018 [M−H]^−^	493.1112, 359.0695, 339.0456, 313.0652, 295.0545, 197.0337, 179.0226, 161.0162, 135.0371	8.47 ± 1.65	6.48 ± 1.58
27.18	29.80	Clinopodic acid I (**25**) [23]	236, 287, 330	718.1534	717.1461	5.30	C_36_H_30_O_16_	717.1423 [M−H]^−^	555.1069, 519.0850, 493.1072, 357.0552, 339.0422, 331.0732, 313.0629, 295.0516, 179.0270	- ^9^	0.32 ± 0.00
27.36	30.48	Salvianolic acid H (**26**) [16,18,19,20,21]	236, 292, 324	538.1111	537.1038	3.81	C_27_H_22_O_12_	537.1018 [M−H]^−^	493.1070, 359.0729, 339.0423, 313.0368, 295.0574, 197.0423, 179.0327, 161.0221, 135.0434	20.37 ± 1.65	6.14 ± 2.58
27.49	29.37	Methyl apigenin deoxyhexosyl hexoside (**27**) [18]	270, 328	592.1792	593.1865	3.35	C_28_H_32_O_14_	593.1845 [M+H]^+^	447.1275, 285.0733	0.19 ± 0.00	1.55 ± 0.01
27.74	31.29	Salvianolic acid E/B/L (**28**) [21]	254, 286, 308, 338	718.1534	717.1461	5.30	C_36_H_30_O_16_	717.1423 [M−H]^−^	537.0811, 519.0733, 493.0994, 357.0477, 339.0348, 321.0287, 313.0602, 295.0503, 197.0385, 185.0176, 179.0279, 135.0390	9.43 ± 0.84	2.00 ± 0.35
28.94	33.41	Salvianolic acid E/B/L (**29**) [21]	232, 288, 332	718.1534	717.1461	5.30	C_36_H_30_O_16_	717.1423 [M−H]^−^	519.0546, 493.0773, 339.0251, 321.0177, 295.0415, 197.0315, 135.0351	4.00 ± 0.76	9.22 ± 1.44
29.53	31.51	Luteolin (**30**) ^4^ [16,18]	252, 268, 348	286.0477	287.0550	8.44	C_15_H_10_O_6_	287.0526 [M+H]^+^	153.0165, 135.0421	0.68 ± 0.08	2.55 ± 0.00
30.49	35.16	Salvianolic acid E/B/L (**31**) [21]	n.r.	718.1534	717.1461	5.30	C_36_H_30_O_16_	717.1423 [M−H]^−^	537.0844, 519.0853, 493.1065, 357.0559, 339.0456, 321.0340, 313.0601, 295.0526, 197.0314, 179.0310, 161.0214, 135.0427	5.20 ± 0.25	0.93 ± 0.00
31.53	34.44	Apigenin (**32**) ^4^ [16,18]	268, 340	270.0528	271.0601	−8.15	C_15_H_10_O_5_	271.0623 [M+H]^+^	153.0174, 119.0484	0.76 ± 0.12	1.41 ± 0.25
-	36.30	Clinopodic acid O (**33**) [23]	n.r.	1076.2223	1075.2150	3.23	C_54_H_43_O_24_	1075.2115 [M−H]^−^	877.1673, 519.0889, 357.0556, 339.0448	-	+
-	42.74	Carnosol (**34**) [18]	n.r.	330.1831	329.1758	4.34	C_20_H_26_O_4_	329.1744 [M−H]^−^	314.1501, 299.1284, 286.1203, 271.0973	+	+
-	45.96	Dehydrocarnosol (**35**) [18]	n.r.	328.1675	327.1602	1.47	C_20_H_24_O_4_	327.1597 [M−H]^−^	299.1616, 284.1257, 269.1048	+	-
		Phenolic acids								165.42	91.58
		Flavonoids								29.22	52.14
		Total identified compounds								194.64	143.72
		Number of identified compounds								33	34

^1^ Rt 1—Retention times recorded using DAD at LC-DAD-MS. ^2^ Rt 2—Retention times recorded using MS at LC-DAD-QTOF-MS/MS. ^3^ References supporting observed fragmentation. ^4^ Identity confirmed using commercial standard compound. ^5^ n.r.—UV spectrum not recorded due to low concentration of compound in extract. ^6^ Phenolic acids were quantified using calibration curve of rosmarinic acid, apigenin flavonoids using calibration curve of apigenin 7-*O*-rutinoside, apigenin 7-*O*-glucuronide or apigenin, vicenin 2 using calibration curve of apigenin 8-*C*-glucoside (vitexin), luteolin flavonoids using calibration curve of luteolin 7-*O*-rutinoside, luteolin 7-*O*-glucuronide or luteolin, hesperetin flavonoid using calibration curve of hesperetin 7-*O*-rutinoside (hesperidin), and hydroxyl kaempferol flavonoid using calibration curve of kaempferol 3-*O*-glucoside (astragalin). ^7^ “+”—detected only using LC-DAD-QTOF-MS/MS. ^8^ tr—trace (<LOQ). ^9^ “-“—not detected.

Previously, the composition of the dry ethyl acetate, methanol and water extracts of *M. nervosa* aerial parts collected in Turkey was analyzed [9]. Like in the current study, the dominant was rosmarinic acid (a marker compound for the Nepetoideae subfamily) [7,9]. Hydroxybenzoic acid, apigenin, caffeic acid and luteolin were also detected [9]. In the current study 30 compounds were detected for the first time for *M. nervosa* (**1–4**, **6**, **8–20**, **22–29**, **31**, **33–35**), including lithospermic acid (**24**) and different salvianolic (**26**, **28**, **29** and **31**) and clinopodic acids (**25** and **33**), suggesting necessity of more studies of samples of different geographic origin. Regarding other *Micromeria* species, salvianolic acids E and B were previously reported for the dry ethanol (95%) extract of *M. imbricata* (Forssk.) C. Chr. aerial parts from Saudi Arabia [26], and one salvianolic acid E, B or L isomer and lithospermic acid were detected in the dry methanol extract of *M. inodora* (Desf.) Benth. aerial parts from Algeria [27]. Also, one salvianolic acid E, B or L isomer, one salvianolic acid H or I isomer, and lithospermic acid were reported for dry methanol (80%) extract of *M. fruticosa* (L.) Druce leaves collected on the West Bank [1]. This name is a synonym for *Clinopodium serpyllifolium* subsp. *fruticosum* (L.) Bräuchler according to Plants of the World Online [6]. Like rosmarinic acid, these compounds and/or their structural analogs are characteristic for various other genera of the Nepetoideae subfamily, such as *Clinopodium* L., *Mentha* L., *Ocimum* L., *Origanum* L., *Salvia* L. and *Satureja* L. [16,18,23,28,29].

It should be noted that the identified phenolic acids, particularly rosmarinic and different salvianolic acids, as well as flavonoids, such as apigenin and luteolin glycosides, are well recognized for their various bioactivities (e.g., antiviral, antioxidant and/or anti-inflammatory activities). In this context, the present study not only expands the knowledge on the phytochemical profile of *M. nervosa*, but also positions this species alongside more extensively studied Nepetoideae taxa as a relevant source of bioactive polyphenols. Moreover, the detection of noted phenolic acids and flavonoids provides a chemical basis for further mechanistic interpretation of the biological effects observed in the continuation of this work.

### 2.2. Phytochemical Profiles of Essential Oils

In the sample_1 essential oil, 31 compounds were identified, accounting for 98.1% of the total oil. Similarly, the analysis of the sample_2 essential oil revealed 30 components, representing 99.4% of the total oil (Table 2; Figures S3 and S4).

In both oils, monoterpenes were dominant. In the sample_1 oil, non-oxygenated and oxygenated monoterpenes were present in equal amounts (45.9% each), while in the sample_2 oil, non-oxygenated monoterpenes (50.2%) were slightly more abundant than oxygenated ones (43.9%). Carvacrol (**59**), a monoterpene phenol, was the major component in both essential oils (29.4% in sample_1 oil and 40.5% in sample_2 oil). The sample_1 oil also contained a significant amount of its isomer thymol (**58**; 12.7%), whereas the sample_2 oil had only a small amount of this compound (0.1%). Both essential oils also contained considerable quantities of *γ*-terpinene (**49**; 14.8 and 32.4%) and *p*-cymene (**45**; 21.1 and 8.6%). Sesquiterpenes were present in smaller amounts (6.2 and 5.3%), with (*E*)-caryophyllene being the most abundant (**62**; 4.0 and 4.5%).

The essential oil previously isolated from the leaves of *M. nervosa* from Tunisia was dominated by a monoterpene *α*-pinene (26.4%) and a sesquiterpene *τ*-cadinol (26.3%) [13], while the most abundant in the essential oil of the aerial parts from Egypt was a sesquiterpene caryophyllene oxide (44.2%) [14]. In the essential oils from Greece analyzed in the present study, *α*-pinene (**37**) and caryophyllene oxide (**65**) were detected in lower amounts (below 1.9%), and *τ*-cadinol was not detected. Similarly, the compounds that dominated our essential oil samples (carvacrol, thymol, *γ*-terpinene and *p*-cymene) were either absent or present in quantities below 0.9% in the essential oils from Tunisia and Egypt [13,14]. However, carvacrol was isolated as the key antimicrobial component from the aerial parts collected in the Palestine area [15]. Oxygenated monoterpenes, such as thymol, carvacrol, pulegone, geranial, borneol and isomenthone, are frequently reported as major constituents of essential oils from *Micromeria* species [7]. Additionally, significant intraspecific variations in composition, including those in thymol and carvacrol content, have been recorded. For instance, in the essential oils from three samples of flowering aerial parts of *M. juliana* (L.) Benth. ex Rchb. collected in Montenegro, carvacrol levels ranged from trace amounts to 18.1%, while thymol levels ranged from traces to 7.3% [31]. The observed variations in *M. nervosa* essential oils suggest the existence of different chemotypes (with potential markers such as carvacrol, thymol, *γ*-terpinene, *p*-cymene, *α*-pinene, *τ*-cadinol and caryophyllene oxide) and provide a solid foundation for further studies on this topic.

Importantly, the abundance of phenolic monoterpenes (carvacrol and thymol) is commonly associated with pronounced bioactivities (e.g., antimicrobial, antioxidant and anticancer effects), indicating that the observed chemical profile may have significant biological relevance [3]. Accordingly, the probable existence of distinct chemotypes within *M. nervosa*, particularly those with enhanced medicinal properties, shifts the focus from a purely descriptive to a functional understanding of the species’ phytochemistry. Moreover, the chemical profile revealed in the present study resembles that of well-known phenolic monoterpene-rich essential oils, such as those of thyme, oregano and summer savory, known for their strong medicinal potential [3].

### 2.3. Antimicrobial Activity

The antimicrobial activity of *M. nervosa* extracts and essential oils was demonstrated against a range of Gram-positive and Gram-negative bacteria, as well as yeast strains (Table 3). In particular, using a microdilution assay, the minimum inhibitory concentration (MIC) values ranging from 0.313 to 10 mg/mL, and the minimum bactericidal/fungicidal concentration (MBC/MFC) values ranging from 0.625 to 10 mg/mL were determined; in a few (i.e., five) cases, MBC values were >10 mg/mL.

Both extracts and essential oils revealed bactericidal effect against the tested bacterial strains, showing MBC/MIC index ≤ 4 (when MBC was available). The most susceptible were Gram-positive strains: *Micrococcus luteus* (MIC = 0.313–1.25 mg/mL, MBC = 1.25–2.5 mg/mL), followed by *Staphylococcus epidermidis* and two *S. aureus* strains (MIC = 0.625–2.5 mg/mL, MBC = 0.625–5 mg/mL); in all four cases, the best activity was revealed for the sample_1 extract. Against *Bacillus cereus*, the growth inhibition was observed at 1.25–2.5 mg/mL; however, bactericidal effects were not recorded at concentrations up to 10 mg/mL, i.e., *B. cereus*, as a spore-forming species, persisted in the presence of the tested extracts/essential oils. The tested Gram-negative bacteria were generally less susceptible compared to the Gram-positive strains. Among them, *Proteus mirabilis* and *Klebsiella pneumoniae* exhibited the highest susceptibility to the tested extracts and essential oils, with MIC values of 2.5 mg/mL and MBC values of 2.5–5 mg/mL. A similar effect (MIC = MBC = 2.5 mg/mL) was revealed only for the essential oils investigated against *Salmonella* Typhimurium and *Escherichia coli*. The observed weaker activity towards *Pseudomonas aeruginosa* is consistent with its robust outer membrane, which limits the penetration of antimicrobial compounds. Given the involvement of *S. aureus*, *E. coli*, *S. epidermidis*, *P. mirabilis* and *K. pneumoniae* in hospital-acquired infections, particularly in immunocompromised patients [32,33], and the role of *S. aureus*, *B. cereus*, *S.* Typhimurium and *E. coli* in foodborne illnesses [34], these results highlight the potential of *M. nervosa* extracts and essential oils for broader antimicrobial applications, including the control of nosocomial and foodborne pathogens.

The essential oils also exhibited notable antifungal properties against all three tested *Candida* species, *C. albicans*, *C. glabrata* and *C. parapsilosis*. The MIC values ranged from 0.313 to 0.625 mg/mL, and MFC values ranged from 0.625 to 1.25 mg/mL. The strongest activity was demonstrated for the sample_1 essential oil against *C. glabrata* and *C. parapsilosis*. These findings are particularly relevant, as the tested *Candida* species are opportunistic pathogens, with *C. albicans* being the leading cause of invasive candidiasis [35]. In addition, *C. albicans* is responsible for common superficial infections such as oral thrush and vaginal candidiasis [33]. On the other hand, *C. glabrata* and *C. parapsilosis* can contaminate food [35,36]. For example, *C. glabrata* has been isolated from food items and wine, likely due to the increasing use of azoles in agriculture and the resulting azole selection pressure [35].

The differences in antimicrobial activity between the extracts and essential oils can be attributed to their distinct chemical compositions. In the performed correlation analysis (Tables S1–S3), the amounts of phenolic acids, such as rosmarinic acid (**21**), lithospermic acid (**24**), and salvianolic acids **26**, **28** and **31** (Pearson’s coefficient, r ≤ −0.96), as well as of luteolin 7-*O*-rutinoside (**11**; r = −0.96) significantly negatively correlated (*p* < 0.05) with activity against *S. aureus*, *M. luteus* and/or *B. cereus* (the higher negative correlation indicates the higher association with activity). On the other hand, the amounts of carvacrol (**59**), (*E*)-caryophyllene (**61**), and monoterpenes **36**, **37**, **41** and **44** (r ≤ −0.96) significantly negatively correlated (*p* < 0.05) with activity on *S.* Typhimurium, *E. coli*, *P. mirabilis* and tested *Candida* species. Similarly, the literature data suggest that the key antimicrobial compounds in the essential oils are carvacrol and thymol [37], while in the extracts, phenolic acids and flavonoids, such as rosmarinic, lithospermic and different salvianolic acids, as well as apigenin, luteolin and their glycosides, play a major role [38,39]. The antimicrobial effects of these compounds are achieved primarily through disruption of cell membranes, leading to increased permeability and loss of cellular contents [37,38]. However, numerous other modes of action were previously revealed. For example, thymol and carvacrol also cause inhibition of efflux pumps, microbial motility and membrane ATPases [37], whereas apigenin and luteolin also affect nucleic acid synthesis [38]. In addition, polyphenols reduce the growth of microorganisms due to inhibition of reactive oxygen species [40]. Although the MIC and MBC/MFC values observed in the current study are relatively high compared to clinically relevant antimicrobials [22], such activity is expected for complex plant extracts and remains biologically relevant, as supported by previous reports on the potent antimicrobial effects of the individual constituents. Our results notably complement previous data on the activity of the dry ethanol, water, ethyl acetate and petroleum ether extracts of *M. nervosa* aerial parts against *S. aureus*, *E. coli*, *P. aeruginosa*, *Proteus vulgaris*, *K. pneumoniae* and *C. albicans*, obtained using the disc diffusion test; the most active was the ethanol extract on *P. vulgaris* and *C. albicans* (27.3 and 27.1 mm) [15]. These findings indicate that *M. nervosa* extracts and essential oils exhibit antibacterial and antifungal activity against clinically relevant pathogens, including foodborne microorganisms. This suggests their potential applicability as complementary antimicrobial agents and as natural preservatives for extending shelf life and improving food safety [2,4].

### 2.4. Cytotoxicity and Anticancer Selectivity

**Figure 1:**
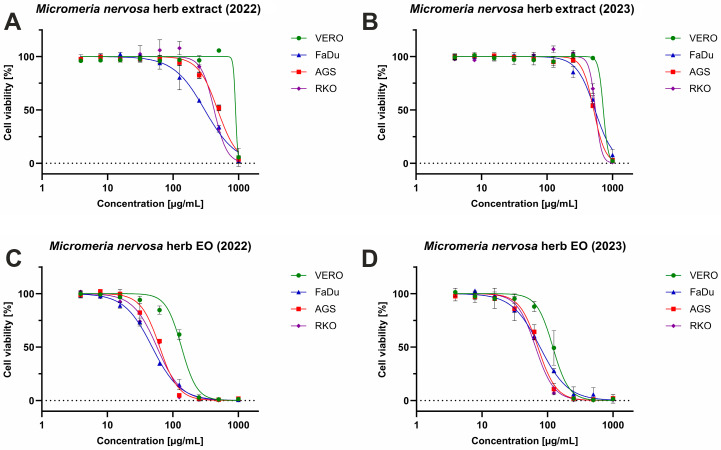
Dose–response effect of investigated *M. nervosa* extracts and essential oils (EO) on a panel of cell lines [2022—sample_1, 2023—sample_2; dose–response cytotoxic effect of sample_1 extract (**A**), sample_2 extract (**B**), sample_1 EO (**C**) and sample_2 EO (**D**) on cell lines].

The cytotoxicity results were evaluated in accordance with the recommendations of the National Cancer Institute (NCI) [41] and previous studies [42,43]. Considering the proposed classification and the results presented in Table 4, it can be concluded that *M. nervosa* extracts were non-cytotoxic (50% cytotoxic concentrations, CC_50_ > 500 μg/mL) to non-cancerous VERO (monkey kidney) cells. No toxicity was also observed when the sample_2 extract was tested on cancer-originating cell lines (AGS cells derived from gastric, FaDu cells from hypopharyngeal, and RKO cells from colorectal cancer). However, the sample_1 extract showed a weak cytotoxic effect (CC_50_ within the range of 200–500 μg/mL) against the same cell lines. Thus, the selectivity indexes (SIs) were also higher in the case of the sample_1 extract compared to the sample_2 extract, suggesting a more selective cytotoxic influence on cancer cell lines. Essential oils obtained from *M. nervosa* were moderately cytotoxic to all cell lines, with statistically significantly higher (*p* < 0.001) cytotoxicity observed for cancer cell lines. The observed SI values for essential oils were noticeably higher for the sample_1 essential oil, but there was a statistically significant difference (*p* < 0.001) in the cytotoxic effect between these two essential oils only towards FaDu cells. Interestingly, when the cytotoxicity of extracts was compared, significant differences were found towards FaDu and RKO cells. The selective impact of *M. nervosa* extracts and essential oils on cancer cell lines can also be seen in dose–response curves in Figure 1.

Previously, dry acetone extract from *M. nervosa* aerial parts, as well as its isolated constituents micromeriol, nervosane, *β*-sitosterol, oleanolic acid and ursolic acid, exhibited cytotoxic activity against three to eight different cancer cell lines, including those derived from colon and gastric cancers, although different from the ones used in our study [11].

Effects observed in the current research can be at least partly attributed to the presence of dominant components of extracts, such as rosmarinic and different salvianolic acids (the sample_1 extract, richer in these compounds, exhibited better activity), and essential oils, primarily thymol and carvacrol, whose cytotoxic effects were previously observed in numerous cancer cells in vitro. Furthermore, for these compounds, there are data suggesting in vivo effectiveness in treatment and prevention of different cancers, including those originating from the gastrointestinal tract, such as gastric and colon cancers [44,45]. For example, chemopreventive effects of rosmarinic acid, thymol and carvacrol on rat colon carcinogenesis were previously demonstrated [46,47,48]. Since essential oils generally showed stronger activity compared to extracts, correlation analysis performed in the current study (Tables S1–S3) revealed significant (*p* < 0.05) negative correlations between carvacrol (**59**) and several other terpenes (**36**, **37**, **41**, **44** and **61**), and activity against AGS and RKO (Pearson’s coefficient, r ≤ −0.95).

Dose–response cytotoxicity curves were also used to calculate non-toxic concentrations of extracts and essential oils used further in antiviral studies. Although the MTT test showed that at 500 μg/mL of both extracts, the VERO cellular viability was comparable to the control cells, microscopic evaluation showed that at this concentration, the cellular morphology was different from that of control cells. Thus, the antiviral experiments were conducted with lower concentrations of extracts (300 μg/mL and lower). For the essential oils, a concentration of 50 μg/mL was selected.

### 2.5. Antiviral Activity

**Figure 2:**
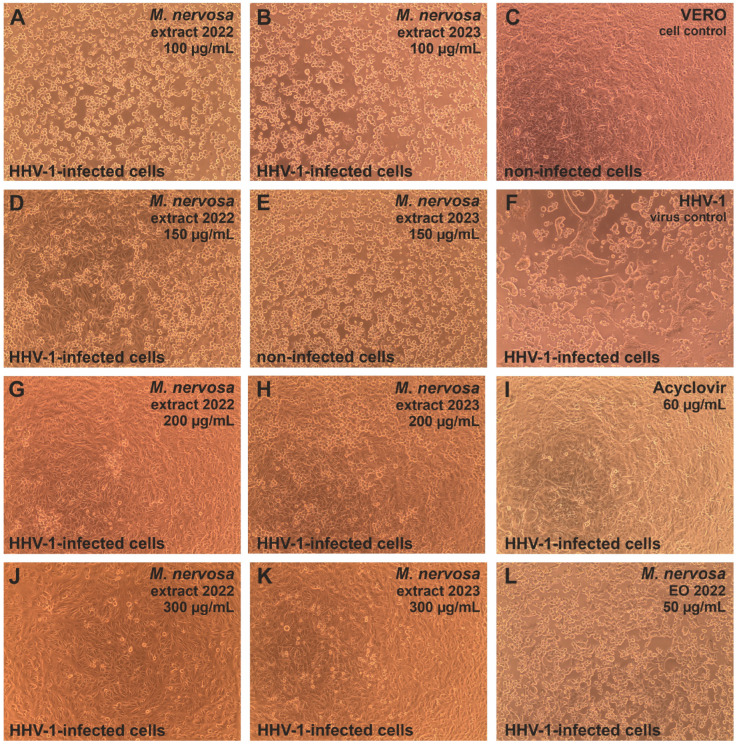
The influence of *M. nervosa* extracts and essential oils on HHV-1-infected VERO cells [(**A**,**B**)—influence of 100 μg/mL extracts from the samples 1 (collected in 2022) and 2 (collected in 2023), respectively, on HHV-1-infected VERO cells; (**C**)—non-infected VERO cells (cell control); (**D**,**E**)—influence of 150 μg/mL extracts from the samples 1 (2022) and 2 (2023), respectively, on HHV-1-infected VERO cells; (**F**)—cytopathic effect induced by HHV-1 infection in VERO cells (HHV-1 virus control); (**G**,**H**)—influence of 200 μg/mL extracts from the samples 1 (2022) and 2 (2023), respectively, on HHV-1-infected VERO cells; (**I**)—antiviral effect of acyclovir 60 μg/mL against HHV-1; (**J**,**K**)—influence of 300 μg/mL extracts from the samples 1 (2022) and 2 (2023), respectively, on HHV-1-infected VERO cells; (**L**)—influence of 50 μg/mL essential oil from the sample_1 (2022) on HHV-1-infected VERO cells; magnification 40×].

Evaluation of the influence of *M. nervosa* natural products on human herpesvirus type 1 (HHV-1) infected VERO cells revealed that the extracts exerted dose–response inhibition of virus-induced cytopathic effect (CPE; Figure 2). For the sample_1 extract, this inhibition was present starting from 150 μg/mL (Figure 2D), while for the sample_2 extract, it was noticeable at the concentration of 200 μg/mL. At 200 μg/mL of the sample_1 extract, the virus-infected cell monolayer resembled the cell control (non-infected VERO cells, Figure 2C), with only a minor fraction of cells showing cell rounding. The acyclovir used as a reference antiviral drug inhibited the CPE formation (Figure 2I). Neither of the tested essential oils influenced the CPE development (Figure 2L); virus-infected cells resembled the virus control (infected, non-treated cells, Figure 2F).

**Figure 3:**
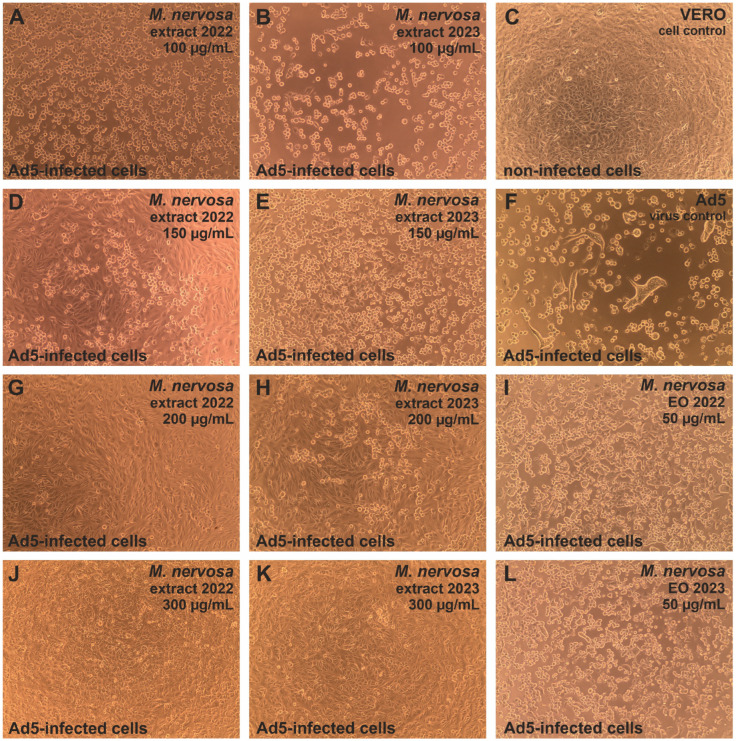
The influence of *M. nervosa* extracts and essential oils on Ad5-infected VERO cells [(**A**,**B**)—influence of 100 μg/mL extracts from the samples 1 (collected in 2022) and 2 (collected in 2023), respectively, on Ad5-infected VERO cells; (**C**)—non-infected VERO cells (cell control); (**D**,**E**)—influence of 150 μg/mL extracts from the samples 1 (2022) and 2 (2023), respectively, on Ad5-infected VERO cells; (**F**)—cytopathic effect induced by Ad5 infection in VERO cells; (**G**,**H**)—influence of 200 μg/mL extracts from the samples 1 (2022) and 2 (2023), respectively, on Ad5-infected VERO cells; (**I**)—influence of 50 μg/mL essential oil from the sample_1 (2022) on Ad5-infected VERO cells; (**J**,**K**)—influence of 300 μg/mL extracts from the samples 1 (2022) and 2 (2023), respectively, on Ad5-infected VERO cells; (**L**)—influence of 50 μg/mL essential oil from the sample_2 (2023) on Ad5-infected VERO cells; magnification 40×].

The extracts also inhibited the development of human adenovirus Ad5-induced CPE in virus-infected VERO cells (Figure 3). The CPE inhibition was observed starting from 150 μg/mL of the sample_1 extract (Figure 3D) and 200 μg/mL of the sample_2 extract (Figure 3H), similarly to the effect observed against HHV-1 infection in VERO cells. Ad5-infected VERO cells treated with the sample_1 extract starting from a concentration of 200 μg/mL (Figure 3G) resembled the cell control (Figure 3C). The virus-infected cells treated with 200 μg/mL of the sample_2 extract showed minor signs of Ad5-CPE, yet at 300 μg/mL, the CPE was abolished. Treatment with the essential oils did not influence the Ad5-CPE (Figure 3I,L).

**Figure 4:**
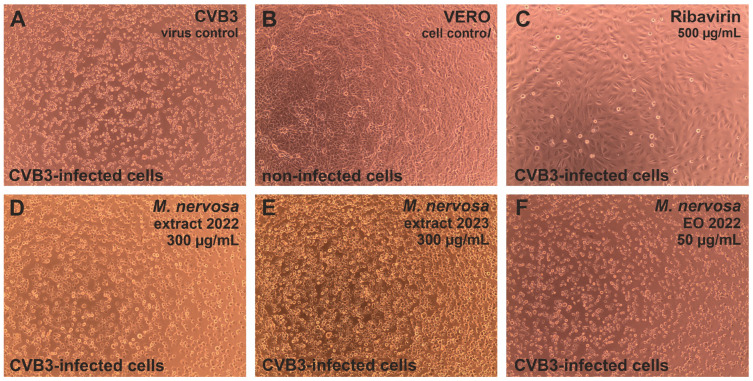
The influence of *M. nervosa* extracts and essential oils on CVB3-infected VERO cells [(**A**)—cytopathic effect induced by CVB3 infection in VERO cells (CVB3 virus control); (**B**)—non-infected VERO cells (cell control); (**C**)—antiviral effect of ribavirin 500 μg/mL against CVB3 in virus-infected VERO cells; (**D**,**E**)—influence of 300 μg/mL extracts from the samples 1 (collected in 2022) and 2 (collected in 2023), respectively, on CVB3-infected VERO cells; (**F**)—influence of 50 μg/mL essential oil from the sample_1 (2022) on CVB3-infected VERO cells; magnification 40×].

The CPE induced by the human coxsackievirus B3 (CVB3), which is an RNA virus in contrast to HHV-1 and Ad5, was not influenced by the tested extracts or essential oil (Figure 4D–F), and treated cells resembled the CVB3 virus control (Figure 4A). The ribavirin 500 μg/mL managed to decrease the CVB3-CPE (Figure 4C) noticeably, but the cellular monolayer was less dense than the one observed in the cell control (Figure 4B).

**Figure 5:**
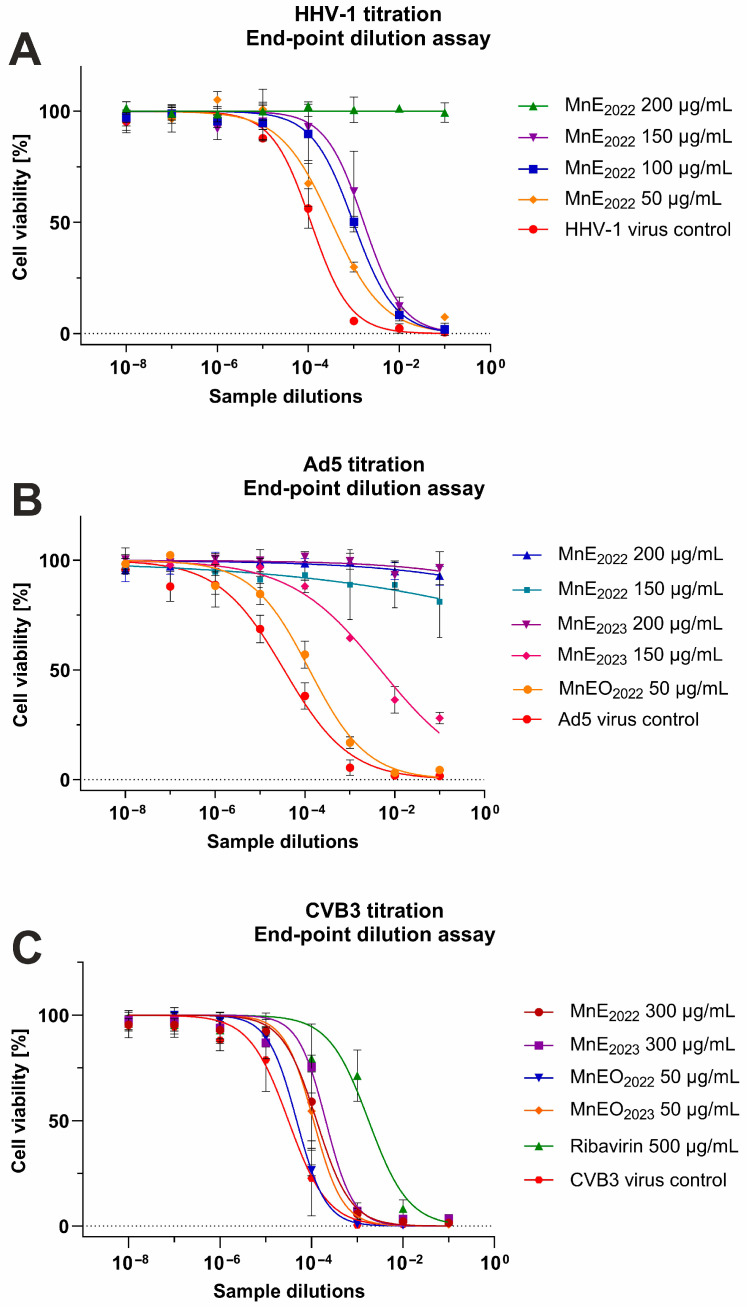
The reduction in HHV-1, Ad5 and CVB3 infectious titers following treatment with tested *M. nervosa* extracts and essential oils from the samples 1 (MnE_2022_, MnEO_2022_) and 2 (MnE_2023_, MnEO_2023_) ((**A**)—dose–response effect of MnE_2022_ on HHV-1 infectious titer; (**B**)—end-point titration of Ad-5 in virus-infected VERO cells treated with the extracts and essential oils; (**C**)—end-point titration of CVB3 in virus-infected VERO cells treated with the extracts and essential oils, as well as ribavirin).

The endpoint dilution assay was done in order to assess the influence of *M. nervosa* extracts and essential oils on the infectivity of the tested viruses. It was found that both extracts not only inhibited the CPE formation in HHV-1-infected VERO cells, but also reduced the infectivity of HHV-1. In fact, the infectious HHV-1 titer in samples treated with the extracts in concentrations above 200 μg/mL could not be established, as it was shown in Figure 5A for the sample_1 extract. Considering the mean HHV-1 viral titer in the virus control (3.9 log), the infectivity reduction was above 3 log, indicating a significant antiviral activity. A similar effect was observed for the acyclovir 60 μg/mL. Lower concentrations of the sample_1 extract—150, 100 and 50 μg/mL reduced the HHV-1 infectious titer by 1.61, 0.93 and 0.46 log, respectively, indicating dose–response inhibition of HHV-1 replication in VERO cells. This effect was markedly lower for the sample_2 extract, which reduced the HHV-1 infectious titer by 1.0, 0.8 and 0.16 log, in the concentration of 150, 100 and 50 μg/mL, respectively. The HHV-1 infectious titer in virus-infected VERO cells treated with both essential oils was comparable to that of the HHV-1 virus control. The mean infectious titer of Ad5 in the virus control was 4.37 log, and taking into account the titration of Ad5 in virus-infected VERO cells treated with both extracts at 200 μg/mL (Figure 5B), the inhibition of Ad5 infectivity was above 4 log, conferring a significant antiviral effect against this virus. The Ad5 titer in samples treated with 150 μg/mL of the sample_1 extract also could not be evaluated, indicating significant inhibition, while in the case of 150 μg/mL of the sample_2 extract, it was reduced by 2.3 log. The reduction in Ad5 infectious titer was below 1 log for the remaining samples. The extracts and essential oils failed to significantly inhibit the infectivity of CVB3, with the reduction rate between 0.18 and 0.82 log (Figure 5C). The ribavirin 500 (Figure 5C) and 250 μg/mL reduced the CVB3 infectious titer by 0.84 and 1.76 log, respectively.

**Figure 6:**
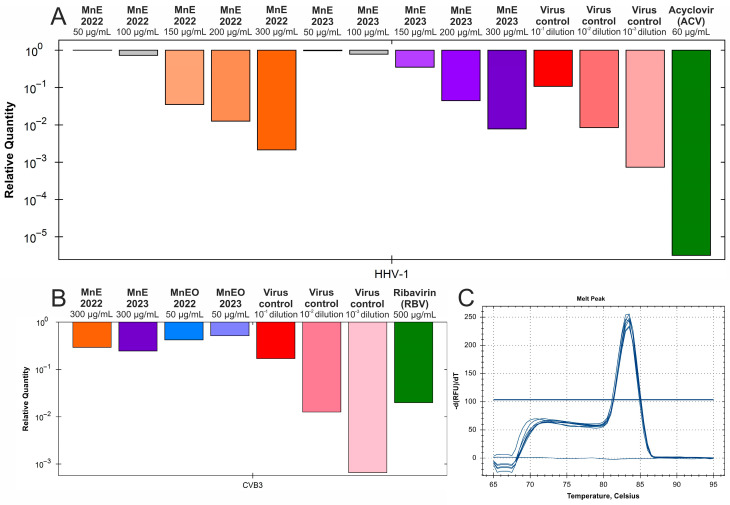
The reduction in HHV-1 and CVB3 viral load following treatment with tested *M. nervosa* extracts and essential oils from the samples 1 (MnE_2022_, MnEO_2022_) and 2 (MnE_2023_, MnEO_2023_) ((**A**)—dose–response effect of the extracts and acyclovir on HHV-1 viral load; (**B**)—the reduction in CVB3 viral load by the extracts, essential oils and ribavirin; (**C**)—DNA melt analysis performed after RT-qPCR amplification of CVB3 RNA).

To gain further insights into the antiviral activity of the investigated extracts and essential oils, the quantification of viral DNA (HHV-1) or RNA (CVB3), often referred to as viral load, was performed. The qPCR amplification of HHV-1 DNA in samples collected from antiviral assays revealed that the sample_1 extract in the concentrations of 300, 200, 150 and 100 μg/mL dose-dependently reduced the HHV-1 viral load by 2.67, 1.89, 1.45 and 0.14 log, respectively (Figure 6A). At the same concentration range, the sample_2 extract reduced the HHV-1 viral load by 2.1 to 0.1 log. Thus, the antiviral activity of *M. nervosa* extracts against HHV-1 is accompanied by reduced amounts of viral DNA, which could, for example, at least partly result from inhibition of its synthesis. Acyclovir, a reference anti-herpesvirus drug, decreased the HHV-1 viral load by 5.5 log. The RT-qPCR amplification of CVB3 RNA in virus-infected cells treated with *M. nervosa* extracts and essential oils revealed only a minor effect on the viral load (Figure 6B). The extracts reduced the CVB3 viral load by 0.53–0.61 log, while the essential oils decreased it by 0.29–0.38 log. The ribavirin at concentrations of 500 and 250 μg/mL reduced the CVB3 viral load by 1.7 and 1.49 log, respectively. The melting curve analysis performed after the qPCR and RT-qPCR amplification confirmed that the same amplicon was present in all samples. The melt curve analysis for CVB3 amplicons is shown in Figure 6C.

The notable antiviral activity of the extracts is consistent with their chemical composition, particularly the high content of phenolic acids. In the correlation analysis, the amounts of phenolic acids, primarily of rosmarinic (**21**) and lithospermic acids (**24**) (Pearson’s coefficient, r ≤ −0.99), significantly negatively correlated (*p* < 0.05) with all antiviral parameters tested (anti-HHV-1 CPE, anti-Ad5 CPE, anti-HHV-1 titer, anti-Ad5 titer and anti-HHV-1 load; Tables S1 and S2). In addition, the amounts of salvianolic acids **26**, **28** and **31** showed the maximum negative Spearman’s correlation coefficient (−1.00) with these parameters (Table S3). Accordingly, the sample_1 extract, which contained higher levels of these compounds, exhibited stronger antiviral effects. Antiviral properties of rosmarinic acid, lithospermic acid and salvianolic acid B have also been previously noticed [49]. Moreover, Astani et al. [50] showed that rosmarinic acid, as well as an aqueous extract of *Melissa officinalis* L. (lemon balm) leaves, rich in this compound, inhibited HHV-1 attachment to host cells in vitro, possibly by interfering with viral surface glycoproteins B and D (gB and gD). However, in the present study, the virus was allowed to attach to host cells prior to treatment; therefore, the observed reductions in CPE and viral titer suggest that extract constituents may also act at post-attachment stages, leading to reduced viral replication (i.e., lower viral load). Altogether, these findings establish a functional link between chemical composition and antiviral activity, moving beyond descriptive phytochemistry toward a more mechanistically oriented interpretation.

It should be noted that among all the bioactivities tested in the current study, the antiviral effects of the extracts against HHV-1 and Ad5 were the most pronounced, as demonstrated by consistent results across multiple experimental approaches. Although the observed effective concentrations were somewhat higher compared to conventional antiviral drugs, such activity is expected for complex plant extracts and remains biologically relevant, particularly in the context of natural product-based therapeutics. The lack of activity against CVB3, in contrast to the pronounced effects against HHV-1 and Ad5, suggests a certain degree of selectivity, potentially related to differences in viral structure and replication mechanisms. From a broader perspective, the present findings position *M. nervosa* as a promising source of antiviral polyphenols, comparable to other well-known Nepetoideae species rich in rosmarinic and related phenolic acids (e.g., lemon balm, *Melissa officinalis*) and support its relevance for pharmaceutical applications. In particular, the activity against DNA viruses such as herpesviruses and adenoviruses highlights the potential of *M. nervosa* extracts for the development of plant-based antiviral formulations targeting common oral infections, as well as other respiratory and gastrointestinal infections [49].

### 2.6. In Silico Evaluation of Binding to HHV-1 Glycoprotein D

As noted in the previous section, Astani et al. [50] demonstrated that rosmarinic acid inhibited HHV-1 attachment to host cells in vitro, and hypothesized that this effect might be mediated through the binding of rosmarinic acid to the viral surface glycoproteins B and D (gB and gD). Attachment of gD primarily to nectin-1 on the host cell membrane is the first step in viral entry via membrane fusion, which ultimately culminates in the fusogenic activity of gB [51]. For gD to bind nectin-1, conformational changes are required, including the displacement of the C-terminus in order to expose amino acid residues 38, 222 and 223 [52]. Accordingly, it was shown that the mutations at positions 222 and 223 (as well as at 215) reduce gD binding efficiency to nectin-1 [51]. Therefore, to gain initial insight into the hypothesis of Astani et al. [50], we performed targeted molecular docking of rosmarinic acid using AutoDock Vina to the region encompassing amino acid residues 38, 215, 222 and 223, as well as the adjacent C-terminal segments of the gD (PDB ID: 2C36). The best-scoring pose showed a binding free energy of −8.3 kcal/mol and involved five hydrogen bonds with residues in this region (Table 5; Figure S5). To verify the robustness of this result, the docking grid was expanded in three subsequent steps, with the third step encompassing the entire protein surface (blind docking). In all cases, the same binding pose was consistently identified as the most favorable, confirming the reliability of the predicted interaction site. This binding site was subsequently used for docking of all other identified extract components present in quantities exceeding 1%, and most of them showed even better docking scores compared to rosmarinic acid (Table 5). For example, salvianolic acid L had the score of −9.9 and formed eight hydrogen bonds (Figure S6), whereas luteolin 7-*O*-glucuronide had the score of −10.9 and formed six hydrogen bonds (Figure S7).

**Figure 7:**
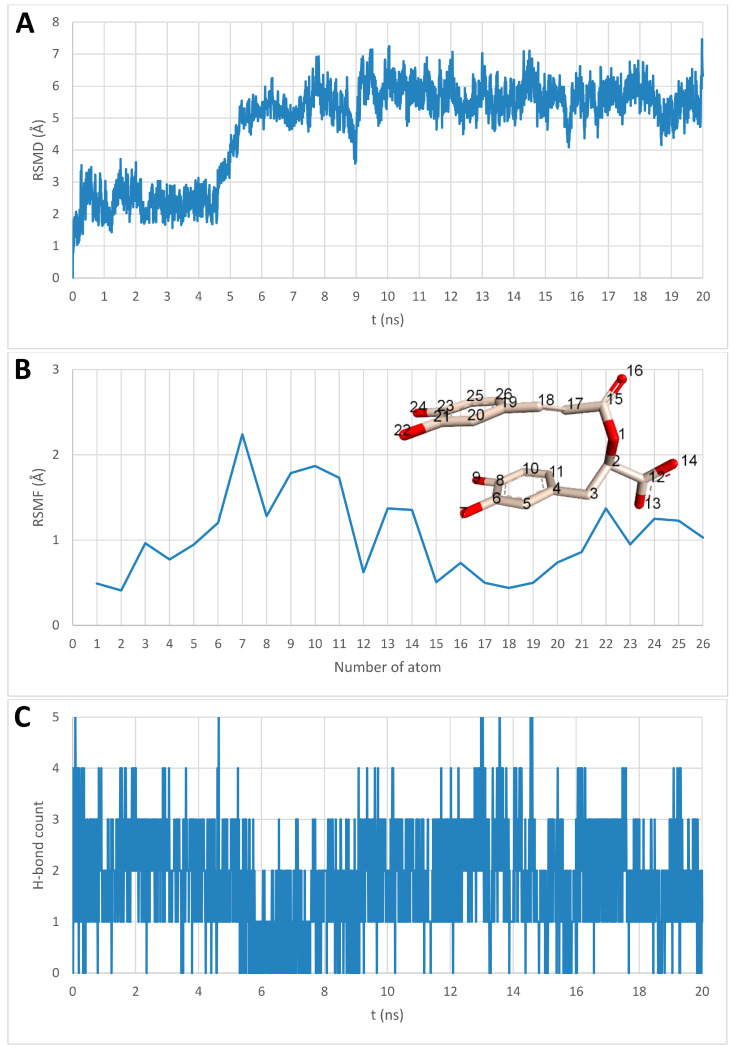
MD analyses: RMSD (**A**), RMSF (**B**) and H-bonding (**C**).

**Figure 8:**
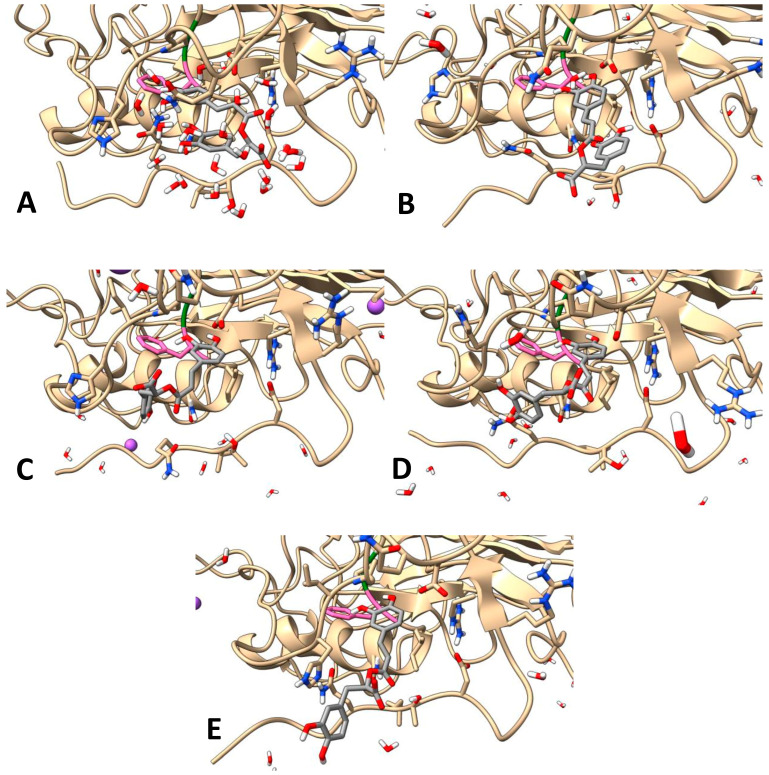
Position of rosmarinic acid (gray) relative to residues 222 (green) and 223 (pink) at 0 ns (**A**), 5 ns (**B**), 10 ns (**C**), 15 ns (**D**) and 20 ns (**E**) of the MD simulation.

To provide a dynamic perspective beyond the static docking results, a molecular dynamics (MD) simulation was performed. Based on available in vitro data [50], rosmarinic acid was selected for an exploratory 20 ns MD simulation of the binding to gD (PDB ID: 2C36) using GROMACS. RMSD analysis indicated positional fluctuations of rosmarinic acid, particularly after 5 ns, yet the ligand remained within the original binding site determined in molecular docking (Figure 7A and Figure 8; Video S1). RMSF analysis revealed that the salvianic acid A unit of rosmarinic acid was more stable, exhibiting lower fluctuations than the caffeic acid unit (Figure 7B). The number of hydrogen bonds varied throughout the trajectory, reaching a maximum of five (Figure 7C), supporting a stable binding mode of rosmarinic acid within the 20 ns timescale.

All of these findings predict a binding mode that could interfere with conformational changes in gD, potentially limiting HHV-1 entry during the initial phase and suggesting one possible mechanism of action of rosmarinic acid. However, as noted previously, since viral adsorption was permitted prior to treatment in the present in vitro model, the reductions in CPE and viral titer point to the ability of extract constituents to affect later stages of the viral life cycle, ultimately leading to decreased replication (i.e., lower viral load). This suggests another possible mechanism distinct from that reported by Astani et al. [50] for rosmarinic acid. Therefore, the current work provides a solid basis for further mechanistic in vitro investigations of both pre- and post-attachment viral inhibition.

### 2.7. Antioxidant Activity

The extracts exhibited strong antioxidant activity (Table 6). The sample_1 extract (Ferric Reducing Antioxidant Power, FRAP value 5.26 mmol Fe^2+^/g) showed slightly higher total antioxidant activity than the sample_2 extract (FRAP value 4.03 mmol Fe^2+^/g). Ascorbic acid (FRAP value 12.73 mmol Fe^2+^/g) was used as a positive control. Similarly, in the DPPH radical scavenging assay, the sample_1 extract (50% free radical scavenging concentration, SC_50_ = 14.40 μg/mL) showed a stronger activity than the sample_2 extract (SC_50_ = 21.83 μg/mL). The essential oils from the sample_1 (SC_50_ = 250.42 μg/mL) and sample_2 (SC_50_ = 494.88 μg/mL) were also tested in the DPPH assay and exhibited interesting radical scavenging activity, though at higher SC_50_ values, which is expected given their different chemical composition. Ascorbic acid (SC_50_ = 3.49 μg/mL) and quercetin (SC_50_ = 4.29 μg/mL) were used as positive controls. The extracts also demonstrated the ability to scavenge hydroxyl radicals in the salicylic acid assay, with the sample_1 extract (SC_50_ = 0.47 mg/mL) again showing somewhat stronger activity than the sample_2 extract (SC_50_ = 0.58 mg/mL). Quercetin (SC_50_ = 0.073 mg/mL) served as a reference compound in this assay. The stronger antioxidant activity of the sample_1 extract is consistent with its higher content of phenolic compounds.

The observed activity of the extracts can be attributed to their dominant polyphenols, such as rosmarinic, lithospermic and different salvianolic acids, as well as apigenin and luteolin glycosides, for which strong antioxidant activity has been extensively reported [39,53]. For example, for rosmarinic acid, significant total antioxidant activity (FRAP value 16.54 mmol Fe^2+^/g) and anti-DPPH activity (SC_50_ = 3.90 μg/mL) were previously demonstrated [22]. Similarly, in the analysis of correlations between the composition and anti-DPPH activity (Tables S1–S3), the strongest negative correlations were found for phenolic acids, for example, for rosmarinic and lithospermic acids (**21** and **24**; Pearson’s coefficient, r ≤ −0.89). In addition, strong negative Spearman’s correlations (−0.95) were found for salvianolic acids **26**, **28** and **31**. The essential oils, despite their different compositions, still exhibited certain radical scavenging properties, likely due to the presence of thymol and carvacrol, compounds known for their antioxidant potential [54].

Previously, dry aqueous, methanol and ethyl acetate extracts of *M. nervosa* aerial parts from Turkey showed significant antioxidant activity in DPPH and ABTS cation (2,2′-azino-bis(3-ethylbenzothiazoline-6-sulfonic acid)) radical scavenging assays and FRAP and CUPRAC (Cupric Reducing Antioxidant Capacity) tests, with the aqueous extract being the most effective [9]. Dry acetone extract of the aerial parts from Egypt exhibited strong DPPH, superoxide and nitric oxide radical scavenging, and lipid peroxidation and xanthine oxidase inhibition, with isolated compounds micromeriol, nervosane, *β*-sitosterol, oleanolic acid and ursolic acid, each contributing to the observed activity [11]. Dry ethanol (70%) extract of the leaves from Tunisia (and its different fractions) also demonstrated antioxidant properties in DPPH radical scavenging and *β*-carotene bleaching tests [12].

The significant antioxidant properties of *M. nervosa* extracts and essential oils complement their antibacterial, anticandidal and/or antiviral activities, supporting potential applications in various industries. In the food industry, these natural sources of antioxidants can enhance preservation by delaying oxidation, thereby extending shelf life and maintaining nutritional quality. They also hold potential for the development of functional foods or even pharmaceuticals aimed at preventing oxidative stress-related conditions. Additionally, in cosmetics, these extracts and essential oils can be utilized in skincare products to protect against oxidative damage and reduce signs of aging [55].

## 3. Materials and Methods

### 3.1. Plant Material

Pleasantly scented flowering aerial parts of *M. nervosa* (perennial plants with lignified stems) were randomly collected from wild populations in August 2022 (summer season, late flowering stage) near the city of Nafplio (37.547944° N, 22.817694° E) (sample_1) and in May 2023 (spring season, early to full flowering phase) on the Methana Peninsula (37.6142099° N, 23.3307551° E) (sample_2), Greece. Voucher specimens are deposited in the Herbarium of the Natural History Museum, Belgrade (BEO) under collector numbers 101812 and 101813. The plant material was dried at room temperature (20–22°C). After drying, leaves and flowers were separated from lignified stems; amounts of dried material: sample_1—leaves and flowers: 36 g, stems: 44 g; sample_2—leaves and flowers: 39 g, stems: 50 g.

The plant binomial was identified and verified according to the taxonomic keys provided in Flora Europaea [56] and Flora Iberica [10]. It should be noted that *M. nervosa* is not yet covered by the available volumes of Flora Hellenica (Lamiaceae pending), nor is it included in the Mountain Flora of Greece, as it is primarily a lowland/coastal species.

### 3.2. Preparation of Dry Hydroethanolic Extracts

Dried and powdered leaves and flowers (5 g), separated from the stems, were extracted with diluted ethanol (70% *w*/*w*, herbal drug-to-solvent ratio = 1:10 *w*/*v*) at room temperature (20–22°C) using bimaceration (two successive 72 h extractions: after the first extraction, the extract was filtered, and the residual material was re-extracted with the same amount of fresh solvent). The filtered extracts were combined, and the solvent was evaporated to dryness using a rotary evaporator (BÜCHI, Flawil, Switzerland); yields: 26.25% *w*/*w* for sample_1 and 27.98% *w*/*w* for sample_2. A preliminary examination of sample_1 showed a significantly lower extract yield from the dried stems (6.12% *w*/*w*; 5 g used for extraction). For this reason, the stems were not used for extraction.

### 3.3. Hydrodistillation of Essential Oils

Yellow essential oils with spicy scent were hydrodistilled from the dried whole leaves and flowers, separated from the stems, using a Clevenger-type apparatus for 2.5 h. The oils were dried over anhydrous sodium sulfate and stored at 4 °C until analysis. The essential oil content was determined in triplicate using 10 g of plant material per hydrodistillation for each sample: 2.91 ± 0.11% *w*/*w* for sample_1 and 3.38 ± 0.11% *w*/*w* for sample_2. The stems were not used for hydrodistillation of essential oils, because a preliminary examination of sample_1 (10 g) showed that the separated dried stems contained very little essential oil (0.02% *w*/*w*).

### 3.4. Chemical Analysis of Dry Hydroethanolic Extracts

#### 3.4.1. LC-DAD-QTOF-MS/MS Analysis

An Agilent 1200 HPLC system, coupled to an Agilent 6210 Quadrupole Time-of-Flight (QTOF) mass spectrometer (Agilent Technologies, Santa Clara, CA, USA), was used in qualitative analysis based on spectral data interpretation. Chromatographic separation was performed on a Phenomenex Gemini C18 column (2 × 100 mm, 3 μm) (Phenomenex, Torrance, CA, USA). The mobile phase consisted of 0.1% formic acid in water (solvent A) and 0.1% formic acid in acetonitrile (solvent B), delivered at a flow rate of 0.2 mL/min (all LC-MS grade). The gradient elution program was as follows: 0–60% B (45 min), 60–95% B (1 min) and kept at 95% B for an additional 9 min (total run time 55 min). The injection volume was 10 μL (concentration of the extract solutions 10 mg/mL). The diode-array detector (DAD) was set at wavelengths of 210, 254, 320 and 366 nm. Detection was performed in both positive and negative electrospray ionization (ESI) modes, with mass spectrometric scanning over the *m*/*z* range of 100–3000. The ion source parameters were configured as follows: nitrogen drying gas at 10 L/min and 275 °C, sheath gas at 12 L/min and 325 °C, nebulizer pressure at 35 psi, capillary voltage at 4000 V, nozzle voltage at 1000 V, fragmentor voltage at 140 V, skimmer voltage at 65 V, and octapole RF peak voltage at 750 V. MS/MS analyses were carried out using collision-induced dissociation energies of 10 and 30 V. Data processing was conducted with MassHunter software version B08.00 (Agilent Technologies). Compounds were characterized based on the obtained tandem mass spectra and by matching the molecular formula, calculated from the measured mass, with data from the KNApSAcK Core System and LOTUS databases [57,58], and in some instances based on the obtained UV spectra.

#### 3.4.2. LC-DAD-MS Analysis

An Agilent 1260 HPLC system, equipped with an Agilent 6130 single quadrupole ESI mass spectrometer, was used in qualitative and quantitative analysis based on employing external standards. The dry extracts were dissolved in a 1:1 (*v*/*v*) mixture of acetonitrile and water at a concentration of 5 mg/mL. Chromatographic separation was carried out using a Zorbax SB-Aq column (Agilent Technologies; 150 × 3.0 mm; 3.5 μm). The mobile phase consisted of 0.1% formic acid (A) and acetonitrile (B) (LC-MS grade), with a gradient elution program as follows: 10–15% B (10 min), 15–25% B (10 min), 25–65% B (15 min), 65–90% B (5 min) and 90–10% B (5 min), at a column temperature of 25°C (total run time 45 min). The injection volume was 5 μL, and the mobile phase flow rate was 0.35 mL/min. DAD was operating at 320 and 350 nm. Mass spectra were acquired in both positive and negative ion modes within the *m*/*z* range of 80–1500 Da, with fragmentor voltage set to 100 and 250 V. Other ion source parameters included a nitrogen flow rate of 10 L/min, a drying gas temperature of 350 °C and a capillary voltage of 3500 V. Identification of compounds was done by matching their retention times, and UV and mass spectra to those of available standards. Quantification of compounds was performed using the external standard method, with commercially available reference substances (Table 7). Quantification was based on peak areas from the DAD chromatograms. Calibration curves were constructed using seven different standard concentrations. The results were presented as mg of compounds per g of dry extracts ± standard deviations resulting from three independent experiments. The limits of detection (LODs) corresponded to the lowest standard concentrations where the signal-to-noise (S/N) ratio reached 3.3, whereas the limits of quantification (LOQs) represented the smallest standard concentrations used in calibration curve construction.

#### 3.4.3. Total Phenolic Content

The total phenolic content was assessed colorimetrically by mixing the extract solutions (0.1 mg/mL in 70% *w*/*w* ethanol; 20 μL) with a 10-fold diluted Folin–Ciocalteu reagent (150 μL) and sodium carbonate solution (60 g/L; 150 μL) in 96-well microtiter plates. After incubating for 90 min, the absorbances were measured at 725 nm using a SPECTROstar Nano microplate reader (BMG Labtech, Ortenberg, Germany). Blank sample was prepared by replacing the extract solutions with 20 μL of 70% *w*/*w* ethanol. The results, presented as the means ± standard deviations from three independent trials, were determined using a gallic acid calibration curve (0.01–0.1 mg/mL) [59].

### 3.5. Chemical Analysis of Essential Oils

Gas chromatography with flame ionization detection and mass spectrometry (GC-FID/MS) analysis was carried out using an Agilent 6890N gas chromatograph (Agilent Technologies, Santa Clara, CA, USA). The system featured a split/splitless injector, a capillary column (Agilent HP-5MS, 30 m × 0.25 mm, 0.25 μm film thickness), an FID detector and an Agilent 5975C mass spectrometer. The injector and FID were maintained at 200 and 300 °C, respectively. Helium was used as the carrier gas at a constant flow rate of 1.0 mL/min. The oven temperature program started at 60 °C, increased to 280 °C at a rate of 3 °C/min and was held at this temperature for 10 min. The injection volume was 1 μL of 1.5% (*v*/*v*) essential oil solution in *n*-hexane, with a split ratio of 1:10. Mass spectrometry was performed in electron ionization (EI) mode at 70 eV. The temperatures of the MSD transfer line, ion source and quadrupole analyzer were set to 250, 230 and 150 °C, respectively. Data were acquired within the *m*/*z* range of 35–550 at a scan speed of 2.83 scans/s. The system was controlled using MSD ChemStation E.01.00.237 software. Linear retention indices (RIs) of the constituents were determined by comparing their retention times to those of *n*-alkanes (C_8_–C_40_) (Fluka, Buchs, Switzerland) analyzed under identical conditions. Compound identification was based on retention indices and mass spectral matching with the NIST/NBS 05 and Wiley 8th edition libraries, as well as the literature [30]. The relative abundance of each compound was calculated from the FID peak areas.

### 3.6. Antimicrobial Activity Testing

The antimicrobial potential of the extracts and essential oils was evaluated using the broth microdilution method in accordance with the European Committee on Antimicrobial Susceptibility Testing (EUCAST) guidelines. Antibacterial activity was tested against selected Gram-positive (*Staphylococcus aureus* ATCC 25923, *S. aureus* ATCC BA1707, *Staphylococcus epidermidis* ATCC 12228, *Micrococcus luteus* ATCC 10240, *Bacillus cereus* ATCC 10876 and *Enterococcus faecalis* ATCC 29212) and Gram-negative bacteria (*Salmonella* Typhimurium ATCC 14028, *Escherichia coli* ATCC 25922, *Proteus mirabilis* ATCC 12453, *Klebsiella pneumoniae* ATCC 13883 and *Pseudomonas aeruginosa* ATCC 9027) using Mueller-Hinton broth as the growth medium. Antifungal activity was assessed against yeast species (*Candida glabrata* ATCC 90030, *C. albicans* ATCC 102231 and *C. parapsilosis* ATCC 22019) in Mueller-Hinton broth supplemented with 2% glucose, which supports yeast growth. Before testing, the extracts and essential oils were dissolved in dimethyl sulfoxide, diluted in the appropriate broth medium, and subsequently subjected to serial two-fold dilutions. The prepared solutions were then transferred into sterile 96-well microtiter plates (Nunc, Roskilde, Denmark) to obtain final concentrations ranging from 10 to 0.313 mg/mL. Microbial inocula were standardized by adjusting fresh cultures in sterile 0.85% NaCl to match a turbidity equivalent to the 0.5 McFarland standard. These inocula were added to the wells, ensuring final concentrations of 5 × 10^5^ CFU/mL for bacterial strains and 5 × 10^4^ CFU/mL for yeast species. Following incubation at 35 °C for 18–20 h, the minimum inhibitory concentration (MIC) was determined based on turbidity, evaluated both visually and by spectrophotometric measurement at 600 nm. MIC was defined as the lowest extract or essential oil concentration inhibiting visible microbial growth. The minimum bactericidal concentration (MBC) and minimum fungicidal concentration (MFC) were determined by transferring 5 μL from each well onto extract/essential oil-free MH agar plates, followed by incubation at 35 °C for 24 h. The lowest concentration that completely suppressed microbial growth was recorded as the MBC/MFC. All tests were performed in triplicate, with the highest recorded value reported. Appropriate controls were included: a dimethyl sulfoxide control (final concentration of 10%, *v*/*v*), a positive control (inoculum without extract or essential oil) and a negative control (extract or essential oil without inoculum).

### 3.7. Cell Culturing, the Evaluation of Cytotoxicity and Anticancer Selectivity

The cytotoxicity of extracts and essential oils was assessed against non-cancerous VERO (ATCC: CCL-81, monkey kidney) cells, as well as selected cell lines derived from gastric (AGS; ATCC: CRL-1739, human gastric adenocarcinoma), hypopharyngeal (FaDu; ATCC: HTB-43, human hypopharyngeal squamous cell carcinoma) and colorectal (RKO; ATCC: CRL-2577, human colon cancer) cancers. The VERO cells were cultured in Dulbecco’s Modified Eagle’s Medium (DMEM; Corning, Tewksbury, MA, USA), while the aforementioned cancer cell lines were maintained in Minimum Essential Medium (MEM; Corning). Additionally, all cell culture media were supplemented with antibiotics (Penicillin-Streptomycin Solution, Corning) and fetal bovine serum (FBS; Corning). The number of passages of all cell lines used in this research did not exceed 20 passages.

Cytotoxicity was assessed utilizing the MTT assay methodology previously delineated by Pecio et al. [60]. In summary, the cells were transferred into 96-well plates and incubated overnight. Subsequently, they were subjected to treatment with serial dilutions of the tested extracts and essential oils in cell media for a duration of 72 h. Following the treatment period, the cell media were discarded, and the monolayers were thoroughly washed with phosphate-buffered saline (PBS; Corning). MTT-supplemented media were introduced, and after a period of 3 h, the formazan product was solubilized. Following an overnight incubation, the absorbance was measured using the Synergy H1 Multi-Mode Microplate Reader (BioTek Instruments, Inc., Winooski, VT, USA) at wavelengths of 540 and 620 nm. The collected data were subsequently exported to GraphPad Prism (version 10.2.0) to ascertain the CC_50_ values (50% cytotoxic concentrations) from the dose–response curves through non-linear regression analysis. Finally, based on the CC_50_ values, selectivity indexes (SI) were calculated to evaluate specificity towards cancer cell lines (SI > 1 indicates anticancer selectivity):

SI = CC_50_VERO/CC_50_Cancer (1)

The collected cytotoxicity data were statistically analyzed using GraphPad Prism (two-way ANOVA, Tukey’s multiple comparisons test).

### 3.8. Antiviral Activity Testing

Antiviral tests were performed against the Human Herpesvirus type 1 (HHV-1, ATCC, VR-260), the Human Adenovirus (Ad5, ATCC, VR-1516) and the Human Coxsackievirus B3 (CVB3, ATCC, VR-30) propagated in VERO cells. The experiments were conducted in the BSL-2 laboratory following previously described experimental procedures [61]. The infectious titers (CCID_50_/mL—50% cell culture infectious concentration) were assessed using an end-point dilution assay. The antiviral tests evaluated the influence of extracts and essential oils on the formation of virus-induced cytopathic effect (CPE) in virus-infected VERO cells, the evaluation of viral infectious titer reduction, and measurement of viral load reduction using qPCR and RT-qPCR.

#### 3.8.1. Influence on Virus-Induced Cytopathic Effect

In summary, VERO cells were cultured in 48-well Falcon plates (clear flat bottom, TC-treated, Corning) and exposed to a suspension of HHV-1, Ad5 or CVB3 (100-fold CCID_50_/mL) in cell media (500 μL/well) for 1 h, with at least two wells remaining uninfected as controls. Following the viral infection, the media was eliminated, the monolayers were rinsed with PBS, and the non-toxic concentrations of extracts and essential oils were diluted in the cell media and subsequently added. Control wells containing uninfected VERO cells (cell control) were provided with media containing 2% FBS, similar to the virus-infected wells that did not receive any treatment (virus control). The plates were incubated until cytopathic effects (CPE) were observed in the virus control wells. Subsequently, the plates were examined using an inverted microscope (CKX41, Olympus Corporation, Tokyo, Japan) equipped with a camera (Moticam 3+, Motic, Hong Kong, China) to assess any inhibition of CPE by the evaluated extracts and essential oils in comparison to the virus control, and the results were adequately documented (Motic Images Plus 2.0, Motic).

#### 3.8.2. Reduction in Viral Infectious Titer

An endpoint dilution assay was applied to evaluate the reduction in viral infectious titer following treatment with extracts and essential oils. The monolayers of VERO cells cultured in 96-well plates were subjected to tenfold dilutions (10^−1^–10^−12^) of samples obtained from the aforementioned antiviral assays and incubated for a duration of 72 h. The plates were examined daily using an inverted microscope (CKX41, Olympus Corporation). Subsequently, the media were removed, and the MTT method, as previously described, was employed to evaluate the viability of cells treated with various sample dilutions [62]. The collected data were utilized to estimate the CCID_50_/mL values using GraphPad Prism software. To assess the extent of antiviral activity, the difference in viral infectious titer (Δlog) between the virus control (logCCID_50_VC) and extracts/essential oils-treated samples (logCCID_50_Mn) was calculated using the formula:

Δlog = logCCID_50_VC − logCCID_50_Mn (2)

The Δlog values were computed for each endpoint dilution assay and presented as mean Δlog. A significant antiviral activity can be reported for samples exhibiting a reduction in virus titer of at least 3 log.

#### 3.8.3. Reduction in Viral Load

The viral loads of HHV-1 and CVB3 were quantified in samples obtained from antiviral assays through the utilization of qPCR (quantitative polymerase chain reaction) and RT-qPCR (reverse transcriptase quantitative polymerase chain reaction), respectively. The DNA of HHV-1 and RNA of CVB3 were isolated using QIAamp DNA Mini Kit (Cat.: 51304 QIAGEN GmbH, Hilden, Germany) and QIAamp Viral RNA Mini Kit (Cat.: 52904 QIAGEN GmbH), respectively. The RNA isolates from CVB3-infected cells underwent one-step RT-qPCR amplification with iTaq Universal SYBR Green One-Step Kit (Cat.: 1725150, Bio-Rad Laboratories, Life Science Group, Hercules, CA, USA) and enterovirus-specific primers (entrinR (5′-GAAACACGGACACCCAAAGTA-3′) and entrinF (5′-CGGCCCCTGAATGCGGCTAA-3′)) on the CFX96 thermal cycler (Bio-Rad Laboratories). The conditions for RT-qPCR amplification were as follows: reverse transcription (50 °C, 10 min), activation of polymerase (95 °C, 1 min), cycling (40 repeats: denaturation (95 °C, 10 s), annealing and synthesis (65 °C, 30 s), fluorescence acquisition), and melting curve analysis (65–95 °C, 0.5 °C increment/5 s). The DNA isolates from anti-HHV-1 assays were subjected to qPCR amplification with SsoAdvanced Universal SYBR Green Supermix (Bio-Rad Laboratories) and primers (UL54F–5′ CGCCAAGAAAATTTCATCGAG 3′, and UL54R–5′ ACATCTTGCACCACGCCAG 3′) on the CFX96 thermal cycler. The qPCR parameters: polymerase activation (98 °C, 3 min); cycling (40 repeats: DNA denaturation (95 °C, 10 s), annealing and synthesis (60 °C, 30 s), fluorescence acquisition); melting curve analysis (65–95 °C). The CVB3 and HHV-1 viral loads in the extracts/essential oils-treated samples were calculated by comparing them with VC (virus control) using the relative quantity (ΔCq) method on CFX Manager™ Dx Software (Bio-Rad Laboratories) [61,62]. The sensitivity of RT-qPCR and qPCR was evaluated by analyzing dilutions (10, 100 and 1000-fold) of virus RNA isolate (CVB3) or DNA isolate (HHV-1).

### 3.9. Molecular Docking Simulations

Components of the extracts present in quantities exceeding 1% were docked onto the HHV-1 host cell-entry glycoprotein D (gD) using AutoDock Vina 1.1.2 [63]. 3D structures of the ligands were retrieved from PubChem. In the case of salvianolic acids B, E and L, only 2D structures were available; therefore, OpenBabel 2.4.1 [64] was employed to generate their 3D conformations. This software was also used to assign the protonation states of the ligands at pH 7.4 (physiological pH). Ligand geometry optimization was performed using the MMFF94 force field in Avogadro 1.2. Non-polar hydrogens were merged, and rotatable bonds were defined using AutoDock Tools 1.5.6 (The Scripps Research Institute). The protein was retrieved from Protein Data Bank (PDB; ID: 2C36) and preprocessed using the Dock Prep function in UCSF ChimeraX 1.10 [65], which involved removal of solvent molecules and uncomplexed ions, as well as adjustment of potentially incomplete side chains using the Dunbrack rotamer library. Polar hydrogens were added, and non-polar hydrogens merged using AutoDock Tools 1.5.6. The grid box was centered at coordinates (x = 54.398, y = 46.422, z = 101.129) with dimensions (x = y = z = 20) and exhaustiveness set to 60. To verify the robustness of the predicted binding site, blind docking was performed using a grid box encompassing the entire protein (coordinates same as above; dimensions: x = 70, y = 70, z = 90). The protein was rigid, while the ligands were flexible. Nine docking modes were generated per ligand, with the lowest-energy pose (calculated using AutoDock Vina scoring function) analyzed. For this purpose, molecular interactions were visualized using Discovery Studio Visualizer 2019 v19.1.0.18287 (BIOVIA, San Diego, CA, USA).

### 3.10. Molecular Dynamics Simulations

The protein 2C36 was complexed with the highest-scoring docking pose of rosmarinic acid and subjected to molecular dynamics (MD) simulations using GROMACS 2025.2 [66]. The CHARMM36m force field was applied to the protein [67], and the ligand was parameterized using the CHARMM General Force Field (CGenFF) [68]. The complex was placed in a dodecahedral box with a minimum solute-box distance of 1.0 nm, solvated with TIP3P water, and neutralized with counterions. Energy minimization was performed using the steepest descent algorithm, followed by system equilibration. Temperature was maintained at 300 K using the V-rescale thermostat and pressure at 1.0 bar using the C-rescale barostat. Production MD was run for 20 ns under periodic boundary conditions. Trajectory analysis focused on the ligand: root mean square deviation (RMSD) was used to monitor its positional stability relative to the protein, while root mean square fluctuation (RMSF) identified its most stable atoms. Hydrogen bonds between ligand and protein were also monitored. Additionally, to validate the global conformational stability of the protein, its radius of gyration (Rg) was calculated throughout the simulation. The Rg remained stable, with an average value of 1.923 ± 0.007 nm. The Rg components along the X, Y and Z axes also showed minor fluctuations (1.400 ± 0.056, 1.671 ± 0.052, and 1.624 ± 0.034 nm, respectively), indicating overall structural stability.

### 3.11. Antioxidant Activity Testing

#### 3.11.1. Total Antioxidant Activity of Dry Hydroethanolic Extracts

The total antioxidant activity was evaluated using the ferric reducing antioxidant power (FRAP) assay. Extracts, dissolved in 70% *w*/*w* ethanol (0.125 mg/mL; 20 μL), were combined with 300 μL of freshly prepared FRAP reagent in 96-well microtiter plates. This reagent was prepared by mixing 25 mL of acetate buffer (300 mM, pH 3.6), 2.5 mL of 2,4,6-tris(2-pyridyl)-s-triazine (TPTZ) solution (10 mM in 40 mM hydrochloric acid) and 2.5 mL of ferric chloride solution (20 mM). After 30 min, absorbances were recorded at 593 nm using a SPECTROstar Nano microplate reader. Blank sample was prepared by replacing the extract solutions with 20 μL of 70% *w*/*w* ethanol in 300 μL of FRAP reagent. Ascorbic acid (0.02 mg/mL) served as a positive control. The results, reported as the means ± standard deviations from three independent trials, were determined using a ferrous sulfate calibration curve (200–1000 mM) [59].

#### 3.11.2. DPPH Scavenging Activity of Dry Hydroethanolic Extracts and Essential Oils

Extracts solutions in 70% *w*/*w* ethanol (0.2 mL; concentrations 6.5–100 μg/mL) or essential oils solutions in absolute ethanol (0.2 mL; concentrations 0.08–2.5 μL/mL) were combined with the 2,2-diphenyl-1-picrylhydrazyl (DPPH) solution in absolute ethanol (0.5 mM; 0.05 mL) in 96-well microplates. After incubation for 30 min, absorbances were recorded at 517 nm, using a SPECTROstar Nano microplate reader, with ethanol (70% *w*/*w* or absolute) serving as the blank. The negative control consisted of the DPPH solution (0.5 mM; 0.05 mL) diluted with absolute ethanol (0.2 mL). Quercetin (concentrations 0.16–10 μg/mL) and ascorbic acid (concentrations 0.375–6.25 μg/mL) were used as positive controls. The scavenging activity S (%) was determined using the formula:

S (%) = 100 × (A_0_ − A_S_)/A_0_ (3)

where A_0_ represents the absorbance of the negative control, and A_S_ denotes the absorbance of the tested sample. Experiments were performed in triplicate. SC_50_ values (concentrations that scavenge 50% free radicals) were calculated through linear regression analysis [59].

#### 3.11.3. Hydroxyl Radical Scavenging Activity of Dry Hydroethanolic Extracts

Extracts, dissolved in 70% *w*/*w* ethanol (0.25 mL; concentrations ranging from 0.625 to 20 mg/mL), were combined with ferrous sulfate (8 mM in 20 μM EDTA; 1.1 mL), hydrogen peroxide (7 mM; 0.5 mL) and salicylic acid (3 mM; 0.75 mL). The mixtures were incubated at 37 °C for 30 min, after which the absorbances of the supernatants were measured at 515 nm, using an Evolution 300 UV/VIS spectrophotometer (Thermo Scientific, Waltham, MA, USA). Blanks were prepared using the same procedure, except that the salicylic acid solution was replaced with 0.75 mL of water. The negative control consisted of methanol (0.25 mL) instead of the sample. Quercetin (concentrations 0.16–10 mg/mL) served as a positive control. The scavenging percentage, S (%), was determined using the same formula as in the DPPH assay, and SC_50_ values were calculated through linear regression analysis [69].

### 3.12. Correlation Analysis Between the Composition and Bioactivity

The analysis was conducted in Statistica 12 (StatSoft Inc., Tulsa, OK, USA). Normality was assessed using the Shapiro–Wilk test. Due to deviations from normality in some variables and a small sample size (*N* = 4), both Pearson’s (parametric) and Spearman’s (nonparametric) correlation coefficients were calculated. Compounds with relative abundance below 1% were excluded to avoid misleading correlations driven solely by their presence in active extracts/essential oils. While Spearman’s correlations were not statistically significant, the directions of correlations generally aligned with Pearson’s, suggesting consistent trends.

## 4. Conclusions

The comprehensive chemical analysis of *Micromeria nervosa* samples from Greece significantly expands the existing knowledge of its composition. Compounds such as lithospermic acid and various salvianolic and clinopodic acids were reported for the first time in this species. The observed variability of essential oil composition in comparison to literature data suggests the existence of distinct chemotypes and underscores the need for further phytochemical and taxonomic investigations across a wider geographic range.

In vitro and computational bioactivity assessments demonstrated considerable antibacterial, antifungal, antiviral, cytotoxic and antioxidant effects, associated with key compounds, including rosmarinic and different salvianolic acids in the extracts and carvacrol in the essential oil, as supported by correlation analysis. These findings highlight the multifunctional potential of *M. nervosa* across various industries. The extracts and essential oil show particular promise in pharmaceutical applications, with the extracts being especially relevant for addressing viral infections and oxidative stress-related disorders, while both extracts and essential oil exhibit potential against nosocomial and foodborne pathogens. The notable antibacterial activity of both the extracts and the essential oil, together with the marked antifungal activity of the essential oils and the strong antioxidant capacity of the extracts, supports their potential application in the food industry, particularly as natural preservatives and in the development of functional foods. In addition, the results obtained support the use of *M. nervosa* as a healthy culinary spice or fragrant herbal tea in daily consumption.

The revealed rich chemical profile and pronounced multifunctional bioactivities position *M. nervosa* as a valuable natural resource, comparable to other well-known species of the Nepetoideae subfamily, thus justifying its further exploration for both scientific and commercial purposes.

## Figures and Tables

**Table 2:** Chemical composition of the investigated *M. nervosa* essential oils.

RI_exp_^1^	RI_lit_^2^	Compound ^3^	Sample_1 (%)	Sample_2 (%)
925	924	*α*-Thujene (**36**)	1.1 ^4^	1.4
933	932	*α*-Pinene (**37**)	1.9	1.3
947	946	Camphene (**38**)	0.8	0.5
972	969	Sabinene (**39**)	tr	0.1
976	974	*β*-Pinene (**40**)	1.2	0.7
989	988	Mycene (**41**)	1.7	1.6
1005	1002	*α*-Phellandrene (**42**)	0.2	0.2
1010	1008	*δ*-3-Carene (**43**)	0.1	0.1
1016	1014	*α*-Terpinene (**44**)	1.9	2.5
1026	1022	*p*-Cymene (**45**)	21.1	8.6
1029	1024	Limonene (**46**)	0.9	0.6
1036	1032	(*Z*)-*β*-Ocimene (**47**)	tr	0.1
1046	1044	(*E*)-*β*-Ocimene (**48**)	0.1	0.1
1059	1054	*γ*-Terpinene (**49**)	14.8	32.4
1066	1065	*cis*-Sabinene hydrate (**50**)	0.2	0.2
1088	1086	Terpinolene (**51**)	0.2	0.1
1099	1095	Linalool (**52**)	1.3	0.5
1165	1165	Borneol (**53**)	1.1	0.6
1177	1174	Terpinene-4-ol (**54**)	0.4	0.2
1185	1179	*p*-Cymene-8-ol (**55**)	0.1	0.1
1243	1232	Thymol, methyl ether (**56**)	0.8	1.6
1253	1241	Carvacrol, methyl ether (**57**)	tr	tr
1296	1289	Thymol (**58**)	12.7	0.1
1309	1298	Carvacrol (**59**)	29.4	40.5
1354	1349	Thymol acetate (**60**)	tr	-
1372	1370	Carvacrol acetate (**61**)	tr	0.1
1421	1417	(*E*)-Caryophyllene (**62**)	4.0	4.5
1453	1452	*α*-Humulene (**63**)	0.2	0.2
1578	1577	Spathulenol (**64**)	0.1	tr
1584	1582	Caryophyllene oxide (**65**)	1.8	0.6
1609	1608	Humulene epoxide II (**66**)	0.1	tr
		Monoterpene hydrocarbons	45.9	50.2
		Oxygenated monoterpenes	45.9	43.9
		Sesquiterpene hydrocarbons	4.2	4.7
		Oxygenated sesquiterpenes	2.0	0.6
		Total identified	98.1	99.4
		Number of identified compounds	31	30

^1^ RI_exp_—retention indices on HP-5MS column relative to C_8_–C_40_ *n*-alkanes. ^2^ RI_lit_—retention indices obtained from the literature [30]. ^3^ Constituents listed in the order of elution on the HP-5MS column. ^4^ Relative area percentage of the compounds obtained from FID area percent data; tr, trace (<0.1%); -, not detected.

**Table 3:** Antimicrobial activity of investigated *M. nervosa* extracts and essential oils expressed as MICs and MBCs/MFCs (mg/mL).

Microorganisms	Sample_1 Extract		Sample_2 Extract		Sample_1 Essential Oil		Sample_2 Essential Oil	
Gram-positive bacteria	MIC	MBC	MIC	MBC	MIC	MBC	MIC	MBC
*S. aureus* ATCC 25923	0.625	0.625	1.25	1.25	2.5	2.5	2.5	2.5
*S. aureus* ATCC BA1707	1.25	1.25	2.5	5	2.5	2.5	2.5	2.5
*S. epidermidis* ATCC 12228	0.625	1.25	1.25	2.5	1.25	2.5	2.5	5
*M. luteus* ATCC 10240	0.313	1.25	0.625	2.5	0.625	2.5	1.25	2.5
*B. cereus* ATCC 10876	1.25	>10	1.25	>10	2.5	>10	2.5	>10
*E. faecalis* ATCC 29212	5	5	10	>10	2.5	5	5	5
Gram-negative bacteria	MIC	MBC	MIC	MBC	MIC	MBC	MIC	MBC
*S.* Typhimurium ATCC 14028	10	10	10	10	2.5	2.5	2.5	2.5
*E. coli* ATCC 25922	10	10	10	10	2.5	2.5	2.5	2.5
*P. mirabilis* ATCC 12453	2.5	5	2.5	5	2.5	2.5	2.5	2.5
*K. pneumoniae* ATCC 13883	2.5	2.5	2.5	2.5	2.5	2.5	2.5	2.5
*P. aeruginosa* ATCC 9027	5	10	10	10	10	10	10	10
Yeasts	MIC	MFC	MIC	MFC	MIC	MFC	MIC	MFC
*C. glabrata* ATCC 90030	10	10	10	10	0.313	0.625	0.625	0.625
*C. albicans* ATCC 102231	5	10	5	10	0.625	0.625	0.625	1.25
*C. parapsilosis* ATCC 22019	1.25	10	5	10	0.313	0.625	0.625	1.25

**Table 4:** Cytotoxicity of investigated *M. nervosa* extracts and essential oils expressed as CC_50_ (μg/mL) and anticancer selectivity (selectivity index, SI).

Extract/Essential Oil	VERO	AGS		FaDu		RKO	
	CC_50_	CC_50_	SI	CC_50_	SI	CC_50_	SI
Sample_1 extract	904.83 ± 25.31	465.43 ± 18.51	1.94	300.60 ± 25.17	3.01	423.68 ± 21.79	2.14
Sample_2 extract	887.58 ± 45.23	514.20 ± 3.99	1.73	536.73 ± 16.27	1.65	537.93 ± 5.63	1.65
Sample_1 essential oil	135.88 ± 4.04	61.92 ± 4.10	2.19	48.48 ± 3.52	2.80	57.14 ± 0.66	2.38
Sample_2 essential oil	128.10 ± 10.47	68.70 ± 5.95	1.86	77.24 ± 2.14	1.66	65.77 ± 1.87	1.95

**Table 5:** Docking scores obtained using AutoDock Vina of the compounds on HHV-1 gD (PDB 2C36) and observed favorable interactions.

Compound	Score ^a^	Observed Favorable Interactions (Residues 23–28 = N-Terminus; Residues 268–307 = C-Terminus) ^a,b^
Rosmarinic acid CID_5281792 ^c^	−8.3	Hydrogen bond: conventional hydrogen bond (5; Gln 27, Asp 30, Arg 36, Asn 227, His 295), carbon hydrogen bond (1; Thr 29). Electrostatic: pi-anion (1; Asp 301). Hydrophobic: pi-sigma (1; Thr 29), pi-alkyl (2; Ile 224, Pro 297). Van der Waals (15; Gln 27, Leu 28, Thr 29, Asp 30, Val 34, Arg 35, Arg 36, Phe 223, Ile 224, Asn 227, His 295, Ile 296, Pro 297, Gln 300, Asp 301).
Lithospermic acid CID_6441498	−9.1	Hydrogen bond: conventional hydrogen bond (8; Gln 27, Arg 35, Arg 36, Arg 36, Ile 224, Asn 227, Ile 296, Gln 300). Hydrophobic: pi-alkyl (1; Arg 35). Van der Waals (17; Gln 27, Leu 28, Asp 30, Val 34, Arg 35, Arg 36, Val 37, Phe 223, Ile 224, Asn 227, His 295, Ile 296, Pro 297, Ser 298, Gln 300, Asp 301, Ala 302).
Salvianolic acid H CID_10052949	−9.5	Hydrogen bond: conventional hydrogen bond (5; Asp 30, Arg 36, Ile 224, Gln 300, Asp 301), carbon hydrogen bond (1; His 295). Electrostatic: charge (1; His 295). Hydrophobic: amide-pi stacked (1; Ile 296), pi-alkyl (1; Pro 297). Van der Waals (22; Gln 27, Leu 28, Thr 29, Asp 30, Pro 31, Pro 32, Gly 33, Val 34, Arg 35, Arg 36, Gln 132, Pro 133, Arg 222, Phe 223, Ile 224, Asn 227, His 295, Ile 296, Pro 297, Ser 298, Gln 300, Asp 301).
Salvianolic acid B CID_6451084	−8.1	Hydrogen bond: conventional hydrogen bond (3; Asp 30, Arg 36, Asp 301). Electrostatic: charge (1; His 295). Hydrophobic: pi-alkyl (2; Ile 224, Pro 297). Van der Waals (19; Gln 27, Leu 28, Thr 29, Asp 30, Pro 31, Pro 32, Gly 33, Val 34, Arg 35, Arg 36, Gln 132, Phe 223, Ile 224, Asn 227, His 295, Ile 296, Pro 297, Gln 300, Asp 301).
Salvianolic acid E CID_86278266	−8.8	Hydrogen bond: conventional hydrogen bond (3; Ile 224, Asn 227, Asn 293). Electrostatic: pi-charge (2; Asp 30, His 295). Hydrophobic: pi-alkyl (1; Arg 36). Van der Waals (19; Leu 25, Asp 26, Gln 27, Leu 28, Thr 29, Asp 30, Arg 35, Arg 36, Gln 210, Phe 223, Ile 224, Asn 227, Asn 293, Trp 294, His 295, Ile 296, Ser 298, Gln 300, Asp 301).
Salvianolic acid L CID_11765414	−9.9	Hydrogen bond: conventional hydrogen bond (8; Gln 27, Gln 27, Gln 210, Ile 224, Asn 227, Asn 293, His 295, Asp 301), carbon hydrogen bond (1; His 295). Electrostatic: charge (1; His 295), pi-charge (2; Asp 30, His 295). Hydrophobic: pi-alkyl (1; Arg 36), pi-pi (1; His 295). Van der Waals (21; Leu 25, Asp 26, Gln 27, Leu 28, Thr 29, Asp 30, Val 34, Arg 35, Arg 36, Gln 210, Phe 223, Ile 224, Asn 227, Asn 293, Trp 294, His 295, Ile 296, Pro 297, Ser 298, Gln 300, Asp 301).
Luteolin CID_5280445	−8.7	Hydrogen bond: conventional hydrogen bond (5; Gln 27, Leu 28, Asp 30, Arg 36, Asn 227). Electrostatic: pi-anion (2; Asp 30, Asp 30). Hydrophobic: pi-alkyl (2; Arg 36, Arg 36), pi-sigma (1; Ile 224). Van der Waals (12; Gln 27, Leu 28, Thr 29, Asp 30, Val 34, Arg 35, Arg 36, Phe 223, Ile 224, Asn 227, Pro 297, Asp 301).
Luteolin 7-*O*- glucuronide CID_5280601	−10.9	Hydrogen bond: conventional hydrogen bond (6; Gln 27, Arg 35, Arg 36, Arg 36, His 295, Gln 300). Electrostatic: pi-anion (1; Asp 30). Hydrophobic: pi-alkyl (4; Arg 35, Arg 35, Arg 36, Arg 36). Van der Waals (16; Gln 27, Thr 29, Asp 30, Val 34, Arg 35, Arg 36, Val 37, Arg 222, Phe 223, Ile 224, His 295, Ile 296, Pro 297, Gln 300, Asp 301, Ala 302).
Luteolin 7-*O*-rutinoside CID_10461109	−10.8	Hydrogen bond: conventional hydrogen bond (6; Gln 27, Gln 27, Arg 35, Arg 36, Arg 36, His 295). Electrostatic: pi-anion (1; Asp 30). Hydrophobic: pi-alkyl (4; Arg 35, Arg 35, Arg 36, Arg 36). Van der Waals (16; Gln 27, Thr 29, Asp 30, Val 34, Arg 35, Arg 36, Val 37, Gln 132, Arg 222, Phe 223, Ile 224, His 295, Ile 296, Pro 297, Gln 300, Asp 301, Ala 302).
Vicenin 2 CID_442664	−10.4	Hydrogen bond: conventional hydrogen bond (6; Gln 27, Thr 29, Val 34, Arg 222, Ile 224, Asp 301), carbon hydrogen bond (3; Leu 28, Asp 30, Phe 223). Hydrophobic: pi-pi (1; His 295). Van der Waals (22; Gln 27, Leu 28, Thr 29, Asp 30, Pro 31, Pro 32, Gly 33, Val 34, Arg 35, Arg 36, Gln 132, Gln 210, Arg 222, Phe 223, Ile 224, Asn 227, His 295, Ile 296, Pro 297, Ser 298, Gln 300, Asp 301).

^a^ Data for the conformation with the best docking score (i.e., the lowest binding free energy, kcal/mol) is presented. ^b^ Interactions recorded using Discovery Studio Visualizer 2019. ^c^ PubChem Compound ID.

**Table 6:** Total antioxidant activity (TAA; FRAP values), DPPH, and/or hydroxyl radical scavenging activity of investigated *M. nervosa* isolates and reference compounds.

Test	Sample_1 Extract	Sample_2 Extract	Sample_1 Essential Oil	Sample_2 Essential Oil	Ascorbic Acid	Quercetin
TAA (mmol Fe^2+^/g of extract)	5.26 ± 0.15	4.03 ± 0.12	n.t. ^1^	n.t.	12.73 ± 0.50	n.t.
DPPH radical scavenging activity (SC_50_, μg/mL)	14.40 ± 0.64	21.83 ± 0.65	250.42 ± 9.83	494.88 ± 24.12	3.49 ± 0.25	4.29 ± 0.16
Hydroxyl radical scavenging activity (SC_50_, mg/mL)	0.47 ± 0.02	0.58 ± 0.05	n.t.	n.t.	n.t.	0.073 ± 0.013

^1^ nt—not tested.

**Table 7:** Data from quantitative analysis of *M. nervosa* dry hydroethanolic extracts.

Compound (Source)	Calibration Curve Equation	R^2^	Linear Range (μg)	LOD (μg)	LOQ (μg)
Apigenin 8-*C*-glucoside (Sigma-Aldrich, Merck, Darmstadt, Germany)	*y* = 3914.1658*x* + 49.9702	0.9958	0.0031–2.50	0.0007	0.0035
Apigenin 7-*O*-rutinoside (HWI Analytik, Ruelzheim, Germany)	*y* = 4609.7559*x* + 13.7098	0.9999	0.0063–2.00	0.0013	0.0055
Apigenin 7-*O*-glucuronide (HWI Analytik)	*y* = 4514.5221*x* − 9.4910	0.9995	0.0063–2.00	0.0015	0.0060
Apigenin (Sigma-Aldrich, Merck)	*y* = 4571.7209*x* − 18.1369	0.9999	0.0031–2.00	0.0009	0.0045
Luteolin 7-*O*-rutinoside (HWI Analytik)	*y* = 7110.2527*x* − 79.8848	0.9996	0.0063–2.00	0.0010	0.0051
Luteolin 7-*O*-glucuronide (HWI Analytik)	*y* = 5020.3480*x* − 2.7332	0.9999	0.0031–2.50	0.0015	0.0061
Luteolin (Sigma-Aldrich, Merck)	*y* = 4614.7997*x* − 3.3609	0.9993	0.0031–2.00	0.0010	0.0050
Quercetin 3-*O*-glucoside (Sigma-Aldrich, Merck)	*y* = 3450.5525*x* + 19.4631	0.9999	0.0008–1.250	0.0002	0.0018
Kaempferol 3-*O*-glucoside (Sigma-Aldrich, Merck)	*y* = 4007.6421*x* + 5.7835	0.9999	0.0063–2.00	0.0015	0.0060
Hesperetin 7-*O*-rutinoside (ChemFaces, Wuhan, China)	*y* = 9402.7000*x* − 18.5320	0.9999	0.0126–2.50	0.0459	0.1835
Rosmarinic acid (Carl Roth, Karlsruhe, Germany)	*y* = 3570.7289*x* + 95.0257	0.9999	0.0006–10.80	0.0003	0.0020

## Data Availability

The original contributions presented in this study are included in the article/Supplementary Materials. Further inquiries can be directed to the corresponding authors.

## Supplementary Materials

The following supporting information can be downloaded at: https://www.mdpi.com/article/10.3390/antibiotics15040374/s1, Figure S1: DAD chromatogram (350 nm) of sample_2 dry hydroethanolic extract recorded on LC-DAD-MS; compound numbers correspond to those listed in Table 1; Figure S2: Base peak chromatograms (BPCs) of sample_2 dry hydroethanolic extract recorded using LC-DAD-QTOF-MS/MS in negative (upper chromatogram) and positive (lower chromatogram) ionization modes; compound numbers correspond to those listed in Table 1 (a peak is labeled only in the chromatogram of the ionization mode in which the compound was detected); Figure S3: FID chromatogram of sample_1 essential oil, with compounds present in amounts greater than 5% marked (compound numbers correspond to those listed in Table 2); Figure S4: Enlarged FID chromatogram of sample_1 essential oil (compound numbers correspond to those listed in Table 2); Table S1: Pearson’s correlation coefficients (r) and color map of correlations; Table S2: Pearson’s correlation coefficients (r) with marked those significant at *p* < 0.05; Table S3: Spearman’s rank order correlations; Figure S5: Binding of rosmarinic acid to gD (PDB ID: 2C36): 3D representation with labeled amino acid residues suspected to be involved in binding to nectin-1 (top); Visualization of ligand-protein interactions using Discovery Studio Visualizer 2019 (bottom); Figure S6: Binding of salvianolic acid L to gD (PDB ID: 2C36): 3D representation with labeled amino acid residues suspected to be involved in binding to nectin-1 (top); Visualization of ligand-protein interactions using Discovery Studio Visualizer 2019 (bottom); Figure S7: Binding of luteolin 7-*O*-glucuronide to gD (PDB ID: 2C36): 3D representation with labeled amino acid residues suspected to be involved in binding to nectin-1 (top); Visualization of ligand-protein interactions using Discovery Studio Visualizer 2019 (bottom); Video S1: Rosmarinic acid_2C36_20 ns MD simulation.

## Abbreviations

The following abbreviations are used in this manuscript:

Ad5	Human adenovirus
AGS	Human gastric adenocarcinoma cells
ATCC	American Type Culture Collection
CC_50_	50% cytotoxic concentration
CCID_50_	50% cell culture infectious concentration
CFU	Colony-forming unit
CPE	Cytopathic effect
CVB3	Human coxsackievirus B3
DAD	Diode-array detector
DMEM	Dulbecco’s modified Eagle’s medium
DPPH	2,2-diphenyl-1-picrylhydrazyl
EI	Electron ionization
ESI	Electrospray ionization
EUCAST	European Committee on Antimicrobial Susceptibility Testing
FaDu	Human hypopharyngeal squamous cell carcinoma cells
FBS	Fetal bovine serum
FID	Flame ionization detector
FRAP	Ferric reducing antioxidant power
GAE	Gallic acid equivalent
gB	Glycoprotein B
GC	Gas chromatography
gD	Glycoprotein D
HHV-1	Human herpesvirus type 1
HPLC	High-performance liquid chromatography
HRMS	High-resolution mass spectrometry
LC	Liquid chromatography
LOD	Limit of detection
LOQ	Limit of quantification
MBC	Minimum bactericidal concentration
MEM	Minimum essential medium
MFC	Minimum fungicidal concentration
MIC	Minimum inhibitory concentration
MS	Mass spectrometry
MTT	3-(4,5-dimethylthiazolyl-2)-2,5-diphenyltetrazolium bromide
NCI	National Cancer Institute
PBS	Phosphate-buffered saline
PDB	Protein Data Bank
qPCR	Quantitative polymerase chain reaction
QTOF	Quadrupole time-of-flight
RKO	Human colon cancer cells
RMSD	Root mean square deviation
RMSF	Root mean square fluctuation
RT-qPCR	Reverse transcriptase quantitative polymerase chain reaction
SC_50_	Concentration that scavenges 50% free radicals
SI	Selectivity index
TPTZ	2,4,6-tris(2-pyridyl)-s-triazine
VERO	Monkey kidney cells
