# Supplementary material for: Integrated Phytochemical Profiling and Bioactivity Evaluation of Micromeria nervosa, with Emphasis on Antimicrobial and Antiviral Properties
Source: Antibiotics (Basel). 2026 Apr 6;15(4):374. doi: 10.3390/antibiotics15040374 (PMC13113600; doi:10.3390/antibiotics15040374)
Supplement: Supplementary file 1 [file antibiotics-15-00374-s001.zip › Figures S1-S7_Tables S1-S3.pdf]

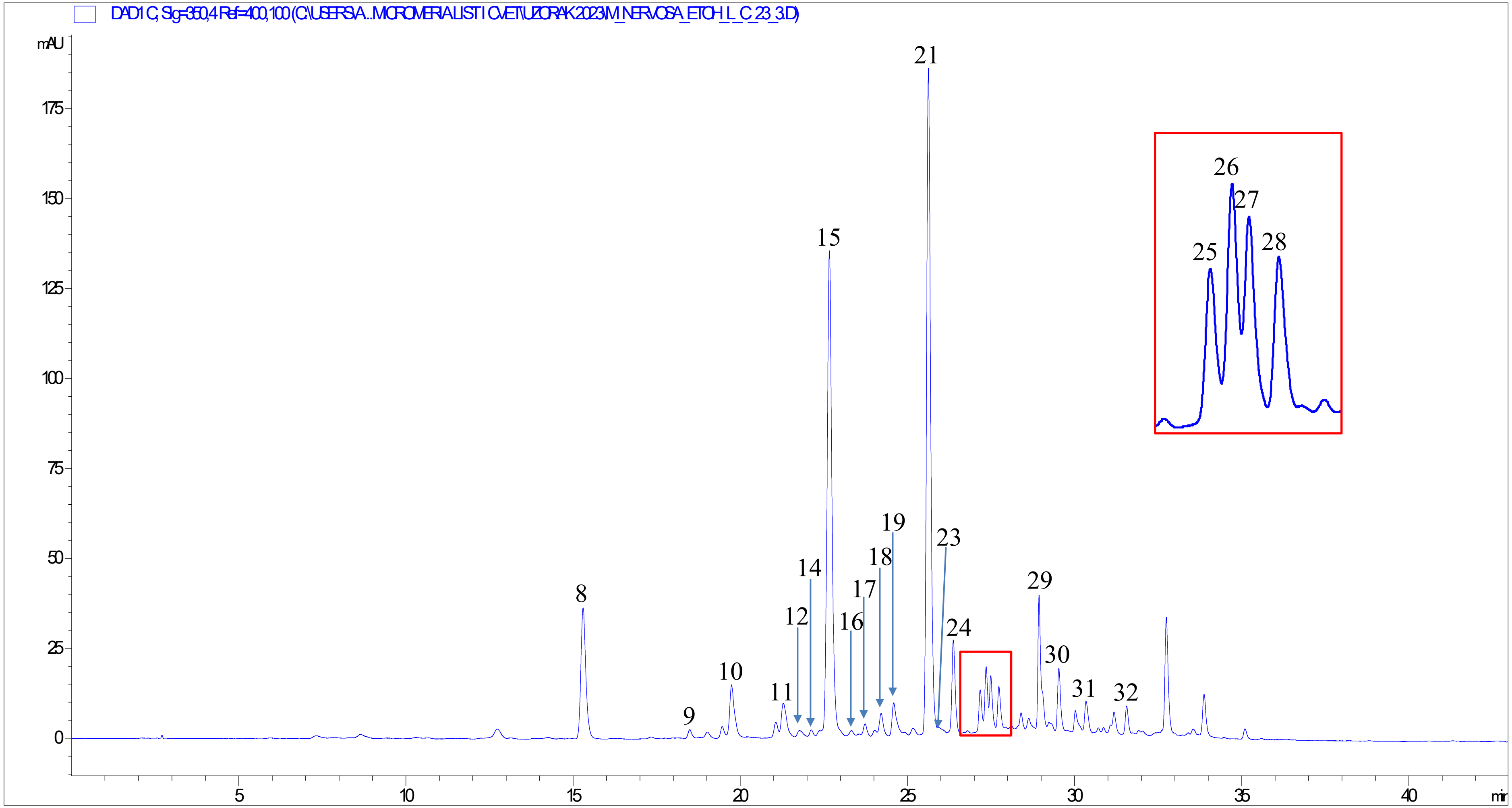

Figure S1. DAD chromatogram (350 nm) of sample_2 dry hydroethanolic extract recorded on LC-DAD-MS; compound numbers correspond to those listed in Table 1.

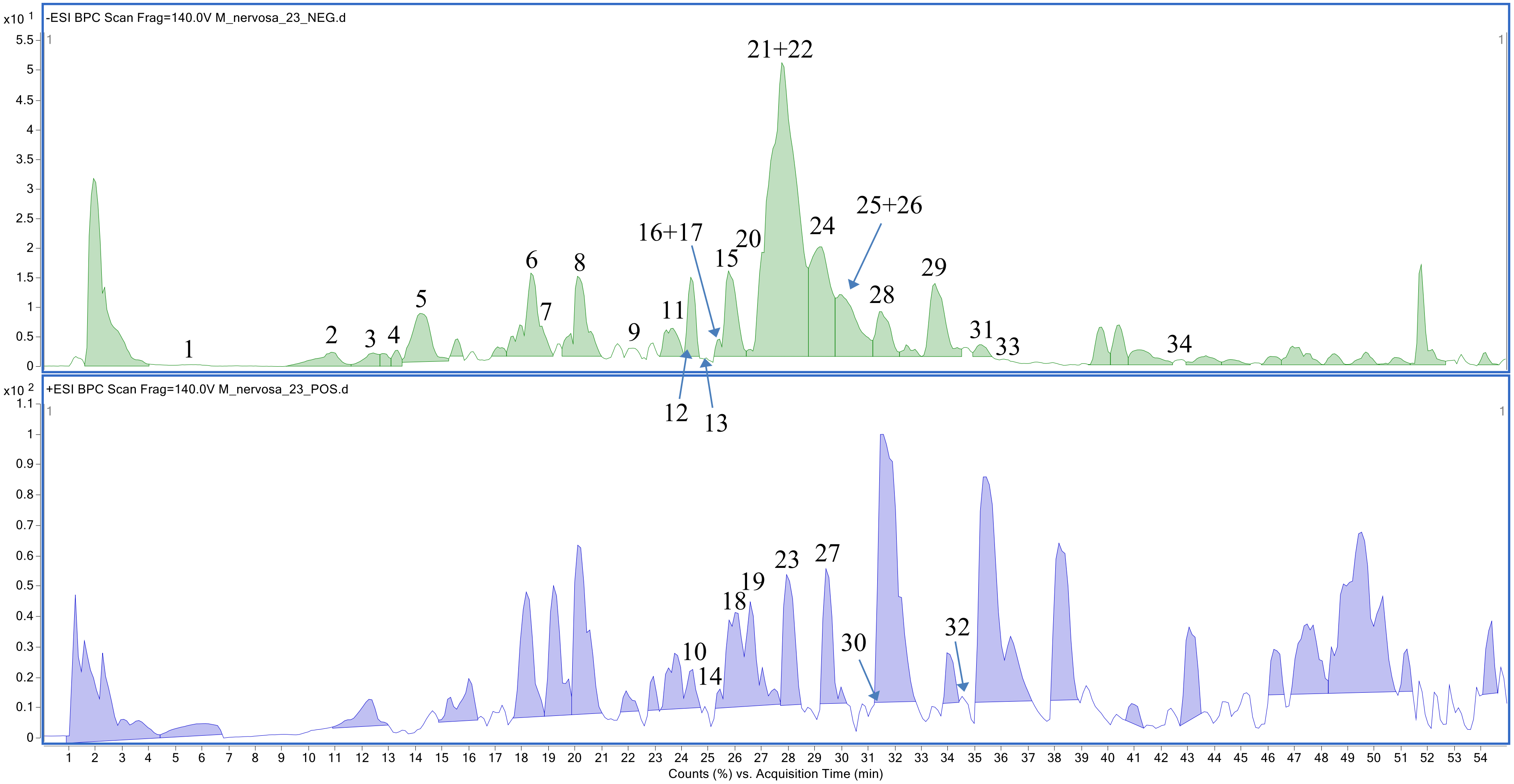

Figure S2. Base peak chromatograms (BPCs) of sample_2 dry hydroethanolic extract recorded using LC-DAD-QTOF-MS/MS in negative (upper chromatogram) and positive (lower chromatogram) ionization modes; compound numbers correspond to those listed in Table 1 (a peak is labeled only in the chromatogram of the ionization mode in which the compound was detected).

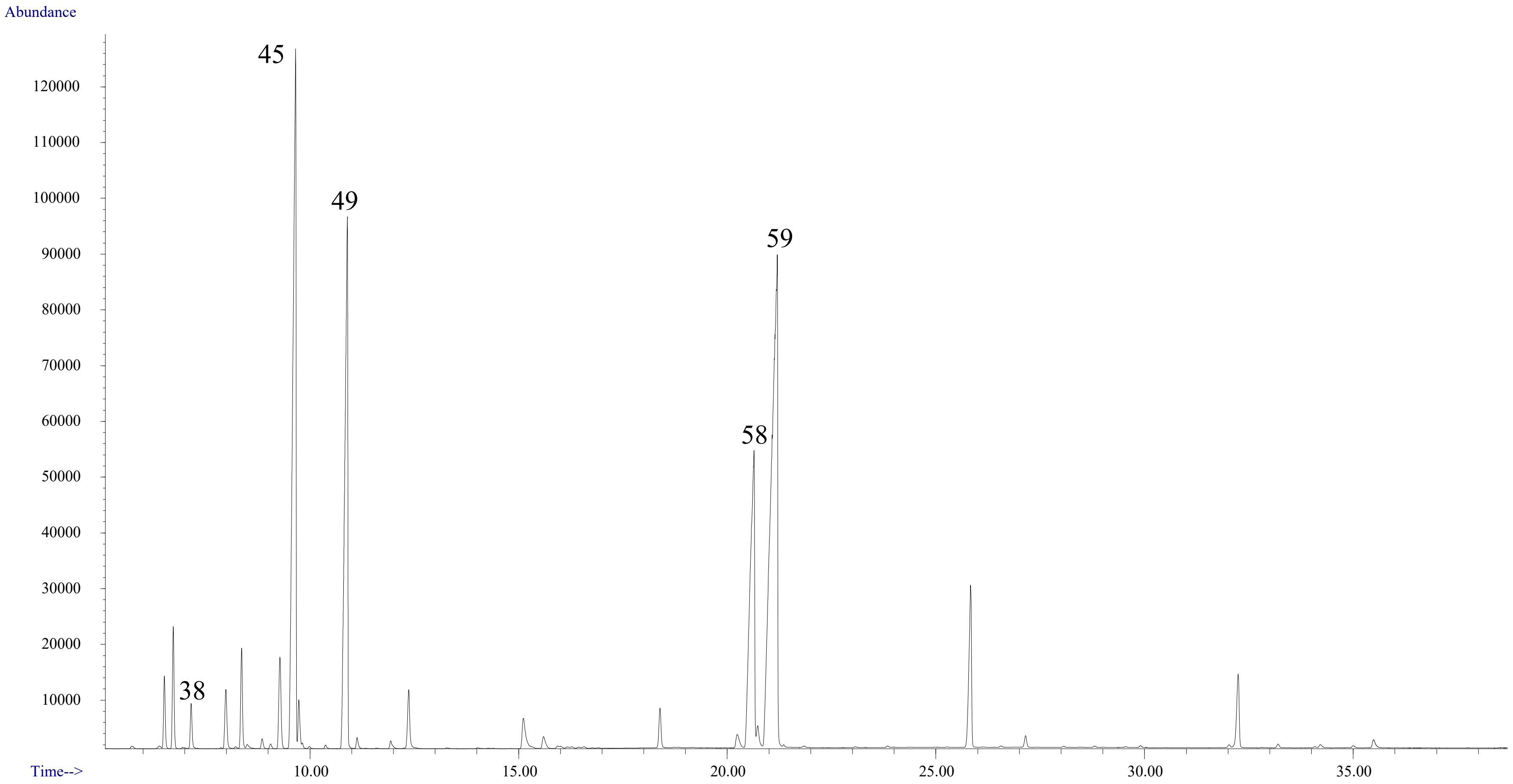

Figure S3. FID chromatogram of sample_1 essential oil, with compounds present in amounts greater than 5% marked (compound numbers correspond to those listed in Table 2).

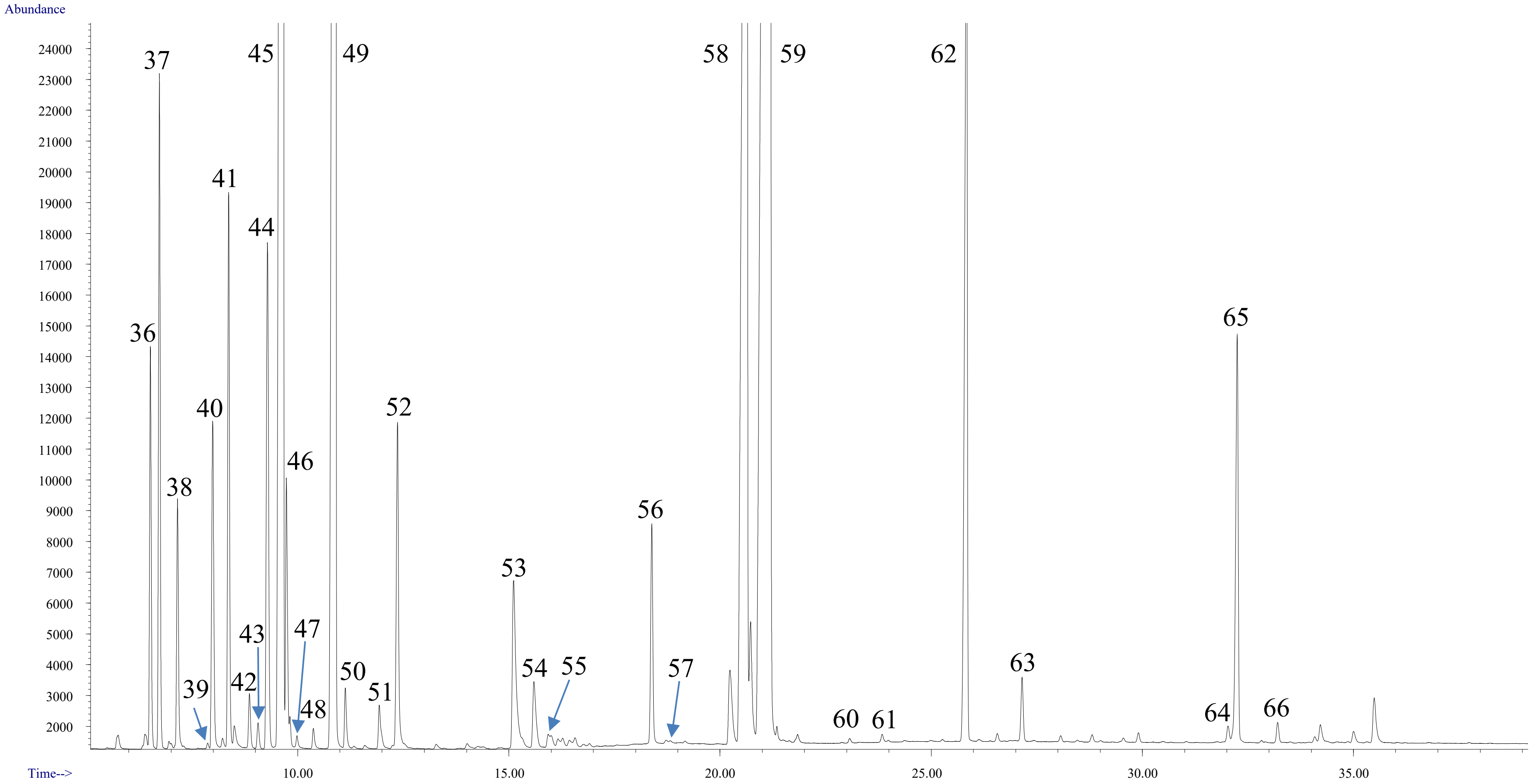

Figure S4. Enlarged FID chromatogram of sample_1 essential oil (compound numbers correspond to those listed in Table 2).

Table S1. Pearson's correlation coefficients (r) and color map of correlations.

Variable	Anti-DPPH activity	*S. aureus* MIC	*S. aureus* MBC	*S. aureus* MIC	*S. aureus* MBC	*S. epidermidis* MIC	*S. epidermidis* MBC	*M. luteus* MIC	*M. luteus* MBC	*B. cereus* MIC	*E. faecalis* MIC	*E. faecalis* MBC	*S.* Typhimurium MIC	*S.* Typhimurium MBC	*E. coli* MIC	*E. coli* MBC	*P. mirabilis* MBC	*P. aeruginosa* MIC
Apigenin 6,8-di-*C*-glucoside (8)	-0.85	-0.82	-0.82	-0.28	0.53	-0.55	-0.55	-0.55	-0.28	-0.95	0.86	0.81	0.95	0.95	0.95	0.95	0.95	-0.28
Hydroxy kaempferol hexuronide (10)	-0.71	-0.61	-0.61	0.02	0.76	-0.36	-0.36	-0.36	0.02	-0.80	0.94	0.95	0.80	0.80	0.80	0.80	0.80	0.02
Luteolin 7-*O*-rutinoside (11)	-0.90	-0.96	-0.96	-0.58	0.22	-0.69	-0.69	-0.69	-0.58	-1.00	0.68	0.57	1.00	1.00	1.00	1.00	1.00	-0.58
Luteolin 7-*O*-glucuronide (15)	-0.72	-0.62	-0.62	0.02	0.76	-0.36	-0.36	-0.36	0.02	-0.81	0.94	0.95	0.81	0.81	0.81	0.81	0.81	0.02
Methyl luteolin deoxyhexosyl hexoside (19)	-0.84	-0.80	-0.80	-0.25	0.56	-0.53	-0.53	-0.53	-0.25	-0.93	0.87	0.83	0.93	0.93	0.93	0.93	0.93	-0.25
Rosmarinic acid (21)	-0.89	-1.00	-1.00	-0.72	0.04	-0.74	-0.74	-0.74	-0.72	-0.98	0.56	0.42	0.98	0.98	0.98	0.98	0.98	-0.72
Lithospermic acid (24)	-0.90	-0.96	-0.96	-0.56	0.25	-0.68	-0.68	-0.68	-0.56	-1.00	0.70	0.60	1.00	1.00	1.00	1.00	1.00	-0.56
Salvianolic acid H (26)	-0.78	-0.97	-0.97	-0.91	-0.30	-0.76	-0.76	-0.76	-0.91	-0.86	0.26	0.08	0.86	0.86	0.86	0.86	0.86	-0.91
Methyl apigenin deoxyhexosyl hexoside (27)	-0.57	-0.41	-0.41	0.25	0.89	-0.20	-0.20	-0.20	0.25	-0.65	0.94	1.00	0.65	0.65	0.65	0.65	0.65	0.25
Salvianolic acid E/B/L (28)	-0.72	-0.93	-0.93	-0.96	-0.42	-0.74	-0.74	-0.74	-0.96	-0.79	0.14	-0.05	0.79	0.79	0.79	0.79	0.79	-0.96
Salvianolic acid E/B/L (29)	-0.72	-0.62	-0.62	0.01	0.76	-0.37	-0.37	-0.37	0.01	-0.81	0.94	0.95	0.81	0.81	0.81	0.81	0.81	0.01
Luteolin (30)	-0.64	-0.51	-0.51	0.14	0.84	-0.28	-0.28	-0.28	0.14	-0.73	0.95	0.98	0.73	0.73	0.73	0.73	0.73	0.14
Salvianolic acid E/B/L (31)	-0.69	-0.91	-0.91	-0.97	-0.46	-0.73	-0.73	-0.73	-0.97	-0.76	0.10	-0.10	0.76	0.76	0.76	0.76	0.76	-0.97
α-Thujene (36)	0.95	0.95	0.95	0.57	-0.23	0.77	0.77	0.77	0.57	0.99	-0.63	-0.57	-0.99	-0.99	-0.99	-0.99	-0.99	0.57
α-Pinene (37)	0.76	0.93	0.93	0.56	-0.22	0.50	0.50	0.50	0.56	0.97	-0.75	-0.56	-0.97	-0.97	-0.97	-0.97	-0.97	0.56
β-Pinene (40)	0.69	0.90	0.90	0.54	-0.22	0.42	0.42	0.42	0.54	0.94	-0.76	-0.54	-0.94	-0.94	-0.94	-0.94	-0.94	0.54
Mycene (41)	0.88	0.96	0.96	0.58	-0.23	0.66	0.66	0.66	0.58	1.00	-0.70	-0.58	-1.00	-1.00	-1.00	-1.00	-1.00	0.58
α-Terpinene (44)	0.97	0.94	0.94	0.57	-0.23	0.80	0.80	0.80	0.57	0.98	-0.61	-0.57	-0.98	-0.98	-0.98	-0.98	-0.98	0.57
p-Cymene (45)	0.55	0.83	0.83	0.50	-0.20	0.26	0.26	0.26	0.50	0.86	-0.76	-0.50	-0.86	-0.86	-0.86	-0.86	-0.86	0.50
γ-Terpinene (49)	1.00	0.85	0.85	0.51	-0.20	0.91	0.91	0.91	0.51	0.88	-0.46	-0.51	-0.88	-0.88	-0.88	-0.88	-0.88	0.51
Linalool (52)	0.53	0.81	0.81	0.49	-0.19	0.24	0.24	0.24	0.49	0.85	-0.76	-0.49	-0.85	-0.85	-0.85	-0.85	-0.85	0.49
Borneol (53)	0.66	0.89	0.89	0.53	-0.21	0.39	0.39	0.39	0.53	0.92	-0.76	-0.53	-0.92	-0.92	-0.92	-0.92	-0.92	0.53
Thymol, methyl ether (56)	1.00	0.87	0.87	0.52	-0.21	0.90	0.90	0.90	0.52	0.90	-0.48	-0.52	-0.90	-0.90	-0.90	-0.90	-0.90	0.52
Thymol (58)	0.17	0.56	0.56	0.34	-0.13	-0.13	-0.13	-0.13	0.34	0.58	-0.67	-0.34	-0.58	-0.58	-0.58	-0.58	-0.58	0.34
Carvacrol (59)	0.97	0.94	0.94	0.56	-0.22	0.81	0.81	0.81	0.56	0.98	-0.60	-0.56	-0.98	-0.98	-0.98	-0.98	-0.98	0.56

(*E*)-Caryophyllene (61)	0.93	0.96	0.96	0.58	-0.23	0.74	0.74	0.74	0.58	1.00	-0.66	-0.58	-1.00	-1.00	-1.00	-1.00	-1.00	0.58
Caryophyllene oxide (64)	0.48	0.79	0.79	0.47	-0.19	0.19	0.19	0.19	0.47	0.82	-0.75	-0.47	-0.82	-0.82	-0.82	-0.82	-0.82	0.47
Phenolic acids	-0.88	-1.00	-1.00	-0.73	0.03	-0.74	-0.74	-0.74	-0.73	-0.98	0.55	0.40	0.98	0.98	0.98	0.98	0.98	-0.73
Flavonoids	-0.77	-0.69	-0.69	-0.09	0.69	-0.43	-0.43	-0.43	-0.09	-0.86	0.92	0.91	0.86	0.86	0.86	0.86	0.86	-0.09
Monoterpene hydrocarbons	0.92	0.96	0.96	0.58	-0.23	0.73	0.73	0.73	0.58	1.00	-0.67	-0.58	-1.00	-1.00	-1.00	-1.00	-1.00	0.58
Oxygeneted monoterpenes	0.88	0.96	0.96	0.58	-0.23	0.67	0.67	0.67	0.58	1.00	-0.70	-0.58	-1.00	-1.00	-1.00	-1.00	-1.00	0.58
Sesquiterpene hydrocarbons	0.93	0.96	0.96	0.58	-0.23	0.74	0.74	0.74	0.58	1.00	-0.66	-0.58	-1.00	-1.00	-1.00	-1.00	-1.00	0.58
Oxygenated sesquiterpenes	0.45	0.77	0.77	0.46	-0.18	0.15	0.15	0.15	0.46	0.80	-0.74	-0.46	-0.80	-0.80	-0.80	-0.80	-0.80	0.46

Color map of correlations											
r ≥	-1	-0.8	-0.6	-0.4	-0.2	0	0.2	0.4	0.6	0.8	1

Table S1. Continued.

Variable	*C. glabrata* MIC	*C. glabrata* MFC	*C. albicans* MIC	*C. albicans* MFC	*C. parapsilosis* MIC	*C. parapsilosis* MFC	VERO CC_{50}	AGS CC_{50}	FaDu CC_{50}	RKO CC_{50}	Anti-HHV1 CPE	Anti-Ad5 CPE	Anti-HHV1 titer	Anti-Ad5 titer	Anti-HHV1 load
Apigenin 6,8-di-*C*-glucoside (8)	0.95	0.95	0.95	0.94	0.90	0.94	0.94	0.97	0.99	0.99	-0.93	-0.93	-0.93	-0.93	-0.93
Hydroxy kaempferol hexuronide (10)	0.80	0.80	0.80	0.80	0.99	0.80	0.79	0.85	0.98	0.90	-0.78	-0.78	-0.78	-0.78	-0.78
Luteolin 7-*O*-rutinoside (11)	1.00	1.00	1.00	1.00	0.70	1.00	1.00	1.00	0.90	0.98	-1.00	-1.00	-1.00	-1.00	-1.00
Luteolin 7-*O*-glucuronide (15)	0.81	0.81	0.81	0.81	0.99	0.81	0.80	0.85	0.98	0.90	-0.78	-0.78	-0.78	-0.78	-0.78
Methyl luteolin deoxyhexosyl hexoside (19)	0.93	0.93	0.93	0.93	0.91	0.93	0.93	0.96	1.00	0.98	-0.92	-0.92	-0.92	-0.92	-0.92
Rosmarinic acid (21)	0.98	0.98	0.98	0.98	0.56	0.98	0.99	0.96	0.81	0.93	-0.99	-0.99	-0.99	-0.99	-0.99
Lithospermic acid (24)	1.00	1.00	1.00	1.00	0.72	1.00	1.00	1.00	0.91	0.99	-1.00	-1.00	-1.00	-1.00	-1.00
Salvianolic acid H (26)	0.86	0.86	0.86	0.86	0.25	0.86	0.87	0.82	0.56	0.75	-0.88	-0.88	-0.88	-0.88	-0.88
Methyl apigenin deoxyhexosyl hexoside (27)	0.65	0.65	0.65	0.65	0.99	0.65	0.63	0.71	0.91	0.78	-0.61	-0.61	-0.62	-0.62	-0.61
Salvianolic acid E/B/L (28)	0.79	0.79	0.79	0.79	0.12	0.79	0.80	0.73	0.45	0.66	-0.81	-0.81	-0.81	-0.81	-0.81
Salvianolic acid E/B/L (29)	0.81	0.81	0.81	0.81	0.99	0.81	0.80	0.85	0.98	0.91	-0.78	-0.78	-0.78	-0.78	-0.78
Luteolin (30)	0.73	0.73	0.73	0.72	1.00	0.72	0.71	0.78	0.95	0.84	-0.70	-0.70	-0.70	-0.70	-0.70

Salvianolic acid E/B/L (31)	0.76	0.76	0.76	0.76	0.07	0.76	0.77	0.70	0.41	0.62	-0.78	-0.78	-0.78	-0.78	-0.78
α-Thujene (36)	-0.99	-0.99	-0.99	-0.98	-0.69	-0.98	-0.99	-0.98	-0.89	-0.97	0.99	0.99	0.99	0.99	0.99
α-Pinene (37)	-0.97	-0.97	-0.97	-0.98	-0.70	-0.98	-0.96	-0.97	-0.89	-0.95	0.97	0.97	0.97	0.97	0.97
β-Pinene (40)	-0.95	-0.94	-0.94	-0.95	-0.68	-0.95	-0.93	-0.94	-0.87	-0.93	0.94	0.94	0.94	0.94	0.94
Mycene (41)	-1.00	-1.00	-1.00	-1.00	-0.71	-1.00	-1.00	-1.00	-0.91	-0.98	1.00	1.00	1.00	1.00	1.00
α-Terpinene (44)	-0.98	-0.98	-0.98	-0.97	-0.68	-0.97	-0.98	-0.98	-0.88	-0.96	0.98	0.98	0.98	0.98	0.98
p-Cymene (45)	-0.87	-0.86	-0.86	-0.88	-0.64	-0.88	-0.86	-0.86	-0.80	-0.85	0.86	0.86	0.86	0.86	0.86
γ-Terpinene (49)	-0.87	-0.88	-0.88	-0.86	-0.60	-0.86	-0.89	-0.88	-0.78	-0.86	0.88	0.88	0.88	0.88	0.88
Linalool (52)	-0.86	-0.85	-0.85	-0.87	-0.63	-0.87	-0.84	-0.85	-0.79	-0.84	0.85	0.85	0.85	0.85	0.85
Borneol (53)	-0.93	-0.92	-0.92	-0.94	-0.67	-0.94	-0.92	-0.92	-0.85	-0.91	0.92	0.92	0.92	0.92	0.92
Thymol, methyl ether (56)	-0.89	-0.90	-0.90	-0.88	-0.61	-0.88	-0.91	-0.90	-0.80	-0.88	0.90	0.90	0.90	0.90	0.90
Thymol (58)	-0.60	-0.58	-0.58	-0.62	-0.46	-0.62	-0.58	-0.59	-0.57	-0.58	0.58	0.58	0.58	0.58	0.58
Carvacrol (59)	-0.97	-0.98	-0.98	-0.96	-0.68	-0.96	-0.98	-0.97	-0.87	-0.95	0.97	0.97	0.97	0.97	0.97
(*E*)-Caryophyllene (61)	-0.99	-1.00	-1.00	-0.99	-0.70	-0.99	-1.00	-0.99	-0.90	-0.98	1.00	1.00	1.00	1.00	1.00
Caryophyllene oxide (64)	-0.83	-0.82	-0.82	-0.84	-0.61	-0.84	-0.81	-0.82	-0.77	-0.81	0.82	0.82	0.82	0.82	0.82
Phenolic acids	0.98	0.98	0.98	0.98	0.55	0.98	0.98	0.96	0.80	0.92	-0.99	-0.99	-0.99	-0.99	-0.99
Flavonoids	0.86	0.86	0.86	0.86	0.97	0.86	0.85	0.90	0.99	0.94	-0.84	-0.84	-0.84	-0.84	-0.84
Monoterpene hydrocarbons	-1.00	-1.00	-1.00	-0.99	-0.70	-0.99	-1.00	-0.99	-0.90	-0.98	1.00	1.00	1.00	1.00	1.00
Oxygeneted monoterpenes	-1.00	-1.00	-1.00	-1.00	-0.71	-1.00	-1.00	-1.00	-0.91	-0.98	1.00	1.00	1.00	1.00	1.00
Sesquiterpene hydrocarbons	-0.99	-1.00	-1.00	-0.99	-0.70	-0.99	-1.00	-0.99	-0.90	-0.98	1.00	1.00	1.00	1.00	1.00
Oxygenated sesquiterpenes	-0.81	-0.80	-0.80	-0.82	-0.60	-0.82	-0.79	-0.80	-0.75	-0.79	0.79	0.79	0.79	0.79	0.79

Table S2. Pearson's correlation coefficients (r) with marked those significant at $p < 0.05$.

Variable	Anti-DPPH activity	*S. aureus* MIC	*S. aureus* MBC	*S. aureus* MIC	*S. aureus* MBC	*S. epidermidis* MIC	*S. epidermidis* MBC	*M. luteus* MIC	*M. luteus* MBC	*B. cereus* MIC	*E. faecalis* MIC	*E. faecalis* MBC	*S.* Typhimurium MIC	*S.* Typhimurium MBC	*E. coli* MIC	*E. coli* MBC	*P. mirabilis* MBC	*P. aeruginosa* MIC
Apigenin 6,8-di-*C*-glucoside (8)	-0.85	-0.82	-0.82	-0.28	0.53	-0.55	-0.55	-0.55	-0.28	-0.95	0.86	0.81	0.95	0.95	0.95	0.95	0.95	-0.28
Hydroxy kaempferol hexuronide (10)	-0.71	-0.61	-0.61	0.02	0.76	-0.36	-0.36	-0.36	0.02	-0.80	0.94	0.95	0.80	0.80	0.80	0.80	0.80	0.02
Luteolin 7-*O*-rutinoside (11)	-0.90	**-0.96**	**-0.96**	-0.58	0.22	-0.69	-0.69	-0.69	-0.58	**-1.00**	0.68	0.57	**1.00**	**1.00**	**1.00**	**1.00**	**1.00**	-0.58
Luteolin 7-*O*-glucuronide (15)	-0.72	-0.62	-0.62	0.02	0.76	-0.36	-0.36	-0.36	0.02	-0.81	0.94	0.95	0.81	0.81	0.81	0.81	0.81	0.02
Methyl luteolin deoxyhexosyl hexoside (19)	-0.84	-0.80	-0.80	-0.25	0.56	-0.53	-0.53	-0.53	-0.25	-0.93	0.87	0.83	0.93	0.93	0.93	0.93	0.93	-0.25
Rosmarinic acid (21)	-0.89	**-1.00**	**-1.00**	-0.72	0.04	-0.74	-0.74	-0.74	-0.72	**-0.98**	0.56	0.42	**0.98**	**0.98**	**0.98**	**0.98**	**0.98**	-0.72
Lithospermic acid (24)	-0.90	**-0.96**	**-0.96**	-0.56	0.25	-0.68	-0.68	-0.68	-0.56	**-1.00**	0.70	0.60	**1.00**	**1.00**	**1.00**	**1.00**	**1.00**	-0.56
Salvianolic acid H (26)	-0.78	**-0.97**	**-0.97**	-0.91	-0.30	-0.76	-0.76	-0.76	-0.91	-0.86	0.26	0.08	0.86	0.86	0.86	0.86	0.86	-0.91
Methyl apigenin deoxyhexosyl hexoside (27)	-0.57	-0.41	-0.41	0.25	0.89	-0.20	-0.20	-0.20	0.25	-0.65	0.94	**1.00**	0.65	0.65	0.65	0.65	0.65	0.25
Salvianolic acid E/B/L (28)	-0.72	-0.93	-0.93	**-0.96**	-0.42	-0.74	-0.74	-0.74	**-0.96**	-0.79	0.14	-0.05	0.79	0.79	0.79	0.79	0.79	**-0.96**
Salvianolic acid E/B/L (29)	-0.72	-0.62	-0.62	0.01	0.76	-0.37	-0.37	-0.37	0.01	-0.81	0.94	0.95	0.81	0.81	0.81	0.81	0.81	0.01
Luteolin (30)	-0.64	-0.51	-0.51	0.14	0.84	-0.28	-0.28	-0.28	0.14	-0.73	0.95	**0.98**	0.73	0.73	0.73	0.73	0.73	0.14
Salvianolic acid E/B/L (31)	-0.69	-0.91	-0.91	**-0.97**	-0.46	-0.73	-0.73	-0.73	**-0.97**	-0.76	0.10	-0.10	0.76	0.76	0.76	0.76	0.76	**-0.97**
α-Thujene (36)	**0.95**	**0.95**	**0.95**	0.57	-0.23	0.77	0.77	0.77	0.57	**0.99**	-0.63	-0.57	**-0.99**	**-0.99**	**-0.99**	**-0.99**	**-0.99**	0.57
α-Pinene (37)	0.76	0.93	0.93	0.56	-0.22	0.50	0.50	0.50	0.56	**0.97**	-0.75	-0.56	**-0.97**	**-0.97**	**-0.97**	**-0.97**	**-0.97**	0.56
β-Pinene (40)	0.69	0.90	0.90	0.54	-0.22	0.42	0.42	0.42	0.54	0.94	-0.76	-0.54	-0.94	-0.94	-0.94	-0.94	-0.94	0.54
Mycene (41)	0.88	**0.96**	**0.96**	0.58	-0.23	0.66	0.66	0.66	0.58	**1.00**	-0.70	-0.58	**-1.00**	**-1.00**	**-1.00**	**-1.00**	**-1.00**	0.58
α-Terpinene (44)	**0.97**	0.94	0.94	0.57	-0.23	0.80	0.80	0.80	0.57	**0.98**	-0.61	-0.57	**-0.98**	**-0.98**	**-0.98**	**-0.98**	**-0.98**	0.57
p-Cymene (45)	0.55	0.83	0.83	0.50	-0.20	0.26	0.26	0.26	0.50	0.86	-0.76	-0.50	-0.86	-0.86	-0.86	-0.86	-0.86	0.50
γ-Terpinene (49)	**1.00**	0.85	0.85	0.51	-0.20	0.91	0.91	0.91	0.51	0.88	-0.46	-0.51	-0.88	-0.88	-0.88	-0.88	-0.88	0.51
Linalool (52)	0.53	0.81	0.81	0.49	-0.19	0.24	0.24	0.24	0.49	0.85	-0.76	-0.49	-0.85	-0.85	-0.85	-0.85	-0.85	0.49
Borneol (53)	0.66	0.89	0.89	0.53	-0.21	0.39	0.39	0.39	0.53	0.92	-0.76	-0.53	-0.92	-0.92	-0.92	-0.92	-0.92	0.53
Thymol, methyl ether (56)	**1.00**	0.87	0.87	0.52	-0.21	0.90	0.90	0.90	0.52	0.90	-0.48	-0.52	-0.90	-0.90	-0.90	-0.90	-0.90	0.52
Thymol (58)	0.17	0.56	0.56	0.34	-0.13	-0.13	-0.13	-0.13	0.34	0.58	-0.67	-0.34	-0.58	-0.58	-0.58	-0.58	-0.58	0.34
Carvacrol (59)	**0.97**	0.94	0.94	0.56	-0.22	0.81	0.81	0.81	0.56	**0.98**	-0.60	-0.56	**-0.98**	**-0.98**	**-0.98**	**-0.98**	**-0.98**	0.56

(*E*)-Caryophyllene (61)	0.93	**0.96**	**0.96**	0.58	-0.23	0.74	0.74	0.74	0.58	**1.00**	-0.66	-0.58	**-1.00**	**-1.00**	**-1.00**	**-1.00**	**-1.00**	0.58
Caryophyllene oxide (64)	0.48	0.79	0.79	0.47	-0.19	0.19	0.19	0.19	0.47	0.82	-0.75	-0.47	-0.82	-0.82	-0.82	-0.82	-0.82	0.47
Phenolic acids	-0.88	**-1.00**	**-1.00**	-0.73	0.03	-0.74	-0.74	-0.74	-0.73	**-0.98**	0.55	0.40	**0.98**	**0.98**	**0.98**	**0.98**	**0.98**	-0.73
Flavonoids	-0.77	-0.69	-0.69	-0.09	0.69	-0.43	-0.43	-0.43	-0.09	-0.86	0.92	0.91	0.86	0.86	0.86	0.86	0.86	-0.09
Monoterpene hydrocarbons	0.92	**0.96**	**0.96**	0.58	-0.23	0.73	0.73	0.73	0.58	**1.00**	-0.67	-0.58	**-1.00**	**-1.00**	**-1.00**	**-1.00**	**-1.00**	0.58
Oxygeneted monoterpenes	0.88	**0.96**	**0.96**	0.58	-0.23	0.67	0.67	0.67	0.58	**1.00**	-0.70	-0.58	**-1.00**	**-1.00**	**-1.00**	**-1.00**	**-1.00**	0.58
Sesquiterpene hydrocarbons	0.93	**0.96**	**0.96**	0.58	-0.23	0.74	0.74	0.74	0.58	**1.00**	-0.66	-0.58	**-1.00**	**-1.00**	**-1.00**	**-1.00**	**-1.00**	0.58
Oxygenated sesquiterpenes	0.45	0.77	0.77	0.46	-0.18	0.15	0.15	0.15	0.46	0.80	-0.74	-0.46	-0.80	-0.80	-0.80	-0.80	-0.80	0.46

Table S2. Continued.

Variable	*C. glabrata* MIC	*C. glabrata* MFC	*C. albicans* MIC	*C. albicans* MFC	*C. parapsilosis* MIC	*C. parapsilosis* MFC	VERO CC_{50}	AGS CC_{50}	FaDu CC_{50}	RKO CC_{50}	Anti-HHV1 CPE	Anti-Ad5 CPE	Anti-HHV1 titer	Anti-Ad5 titer	Anti-HHV1 load
Apigenin 6,8-di-*C*-glucoside (8)	0.95	0.95	0.95	0.94	0.90	0.94	0.94	**0.97**	**0.99**	**0.99**	-0.93	-0.93	-0.93	-0.93	-0.93
Hydroxy kaempferol hexuronide (10)	0.80	0.80	0.80	0.80	**0.99**	0.80	0.79	0.85	**0.98**	0.90	-0.78	-0.78	-0.78	-0.78	-0.78
Luteolin 7-*O*-rutinoside (11)	**1.00**	**1.00**	**1.00**	**1.00**	0.70	**1.00**	**1.00**	**1.00**	0.90	**0.98**	**-1.00**	**-1.00**	**-1.00**	**-1.00**	**-1.00**
Luteolin 7-*O*-glucuronide (15)	0.81	0.81	0.81	0.81	**0.99**	0.81	0.80	0.85	**0.98**	0.90	-0.78	-0.78	-0.78	-0.78	-0.78
Methyl luteolin deoxyhexosyl hexoside (19)	0.93	0.93	0.93	0.93	0.91	0.93	0.93	**0.96**	**1.00**	**0.98**	-0.92	-0.92	-0.92	-0.92	-0.92
Rosmarinic acid (21)	**0.98**	**0.98**	**0.98**	**0.98**	0.56	**0.98**	**0.99**	**0.96**	0.81	0.93	**-0.99**	**-0.99**	**-0.99**	**-0.99**	**-0.99**
Lithospermic acid (24)	**1.00**	**1.00**	**1.00**	**1.00**	0.72	**1.00**	**1.00**	**1.00**	0.91	**0.99**	**-1.00**	**-1.00**	**-1.00**	**-1.00**	**-1.00**
Salvianolic acid H (26)	0.86	0.86	0.86	0.86	0.25	0.86	0.87	0.82	0.56	0.75	-0.88	-0.88	-0.88	-0.88	-0.88
Methyl apigenin deoxyhexosyl hexoside (27)	0.65	0.65	0.65	0.65	**0.99**	0.65	0.63	0.71	0.91	0.78	-0.61	-0.61	-0.62	-0.62	-0.61
Salvianolic acid E/B/L (28)	0.79	0.79	0.79	0.79	0.12	0.79	0.80	0.73	0.45	0.66	-0.81	-0.81	-0.81	-0.81	-0.81
Salvianolic acid E/B/L (29)	0.81	0.81	0.81	0.81	**0.99**	0.81	0.80	0.85	**0.98**	0.91	-0.78	-0.78	-0.78	-0.78	-0.78
Luteolin (30)	0.73	0.73	0.73	0.72	**1.00**	0.72	0.71	0.78	0.95	0.84	-0.70	-0.70	-0.70	-0.70	-0.70
Salvianolic acid E/B/L (31)	0.76	0.76	0.76	0.76	0.07	0.76	0.77	0.70	0.41	0.62	-0.78	-0.78	-0.78	-0.78	-0.78
α-Thujene (36)	**-0.99**	**-0.99**	**-0.99**	**-0.98**	-0.69	**-0.98**	**-0.99**	**-0.98**	-0.89	**-0.97**	**0.99**	**0.99**	**0.99**	**0.99**	**0.99**
α-Pinene (37)	**-0.97**	**-0.97**	**-0.97**	**-0.98**	-0.70	**-0.98**	**-0.96**	**-0.97**	-0.89	**-0.95**	**0.97**	**0.97**	**0.97**	**0.97**	**0.97**
β-Pinene (40)	-0.95	-0.94	-0.94	**-0.95**	-0.68	**-0.95**	-0.93	-0.94	-0.87	-0.93	0.94	0.94	0.94	0.94	0.94

Mycene (41)	**-1.00**	**-1.00**	**-1.00**	**-1.00**	-0.71	**-1.00**	**-1.00**	**-1.00**	-0.91	**-0.98**	**1.00**	**1.00**	**1.00**	**1.00**	**1.00**
α-Terpinene (44)	**-0.98**	**-0.98**	**-0.98**	**-0.97**	-0.68	**-0.97**	**-0.98**	**-0.98**	-0.88	**-0.96**	**0.98**	**0.98**	**0.98**	**0.98**	**0.98**
p-Cymene (45)	-0.87	-0.86	-0.86	-0.88	-0.64	-0.88	-0.86	-0.86	-0.80	-0.85	0.86	0.86	0.86	0.86	0.86
γ-Terpinene (49)	-0.87	-0.88	-0.88	-0.86	-0.60	-0.86	-0.89	-0.88	-0.78	-0.86	0.88	0.88	0.88	0.88	0.88
Linalool (52)	-0.86	-0.85	-0.85	-0.87	-0.63	-0.87	-0.84	-0.85	-0.79	-0.84	0.85	0.85	0.85	0.85	0.85
Borneol (53)	-0.93	-0.92	-0.92	-0.94	-0.67	-0.94	-0.92	-0.92	-0.85	-0.91	0.92	0.92	0.92	0.92	0.92
Thymol, methyl ether (56)	-0.89	-0.90	-0.90	-0.88	-0.61	-0.88	-0.91	-0.90	-0.80	-0.88	0.90	0.90	0.90	0.90	0.90
Thymol (58)	-0.60	-0.58	-0.58	-0.62	-0.46	-0.62	-0.58	-0.59	-0.57	-0.58	0.58	0.58	0.58	0.58	0.58
Carvacrol (59)	**-0.97**	**-0.98**	**-0.98**	**-0.96**	-0.68	**-0.96**	**-0.98**	**-0.97**	-0.87	**-0.95**	**0.97**	**0.97**	**0.97**	**0.97**	**0.97**
(*E*)-Caryophyllene (61)	**-0.99**	**-1.00**	**-1.00**	**-0.99**	-0.70	**-0.99**	**-1.00**	**-0.99**	-0.90	**-0.98**	**1.00**	**1.00**	**1.00**	**1.00**	**1.00**
Caryophyllene oxide (64)	-0.83	-0.82	-0.82	-0.84	-0.61	-0.84	-0.81	-0.82	-0.77	-0.81	0.82	0.82	0.82	0.82	0.82
Phenolic acids	**0.98**	**0.98**	**0.98**	**0.98**	0.55	**0.98**	**0.98**	**0.96**	0.80	0.92	**-0.99**	**-0.99**	**-0.99**	**-0.99**	**-0.99**
Flavonoids	0.86	0.86	0.86	0.86	**0.97**	0.86	0.85	0.90	**0.99**	0.94	-0.84	-0.84	-0.84	-0.84	-0.84
Monoterpene hydrocarbons	**-1.00**	**-1.00**	**-1.00**	**-0.99**	-0.70	**-0.99**	**-1.00**	**-0.99**	-0.90	**-0.98**	**1.00**	**1.00**	**1.00**	**1.00**	**1.00**
Oxygeneted monoterpenes	**-1.00**	**-1.00**	**-1.00**	**-1.00**	-0.71	**-1.00**	**-1.00**	**-1.00**	-0.91	**-0.98**	**1.00**	**1.00**	**1.00**	**1.00**	**1.00**
Sesquiterpene hydrocarbons	**-0.99**	**-1.00**	**-1.00**	**-0.99**	-0.70	**-0.99**	**-1.00**	**-0.99**	-0.90	**-0.98**	**1.00**	**1.00**	**1.00**	**1.00**	**1.00**
Oxygenated sesquiterpenes	-0.81	-0.80	-0.80	-0.82	-0.60	-0.82	-0.79	-0.80	-0.75	-0.79	0.79	0.79	0.79	0.79	0.79

Table S3. Spearman's rank order correlations.

Variable	Anti-DPPH activity	*S. aureus* MIC	*S. aureus* MBC	*S. aureus* MIC	*S. aureus* MBC	*S. epidermidis* MIC	*S. epidermidis* MBC	*M. luteus* MIC	*M. luteus* MBC	*B. cereus* MIC	*E. faecalis* MIC	*E. faecalis* MBC	*S.* Typhimurium MIC	*S.* Typhimurium MBC	*E. coli* MIC	*E. coli* MBC	*P. mirabilis* MBC	*P. aeruginosa* MIC
Apigenin 6,8-di-*C*-glucoside (8)	-0.74	-0.78	-0.78	-0.27	0.33	-0.50	-0.50	-0.50	-0.27	-0.94	0.83	0.82	0.94	0.94	0.94	0.94	0.94	-0.27
Hydroxy kaempferol hexuronide (10)	-0.74	-0.78	-0.78	-0.27	0.33	-0.50	-0.50	-0.50	-0.27	-0.94	0.83	0.82	0.94	0.94	0.94	0.94	0.94	-0.27
Luteolin 7-*O*-rutinoside (11)	-0.95	-1.00	-1.00	-0.82	-0.33	-0.83	-0.83	-0.83	-0.82	-0.94	0.50	0.27	0.94	0.94	0.94	0.94	0.94	-0.82
Luteolin 7-*O*-glucuronide (15)	-0.74	-0.78	-0.78	-0.27	0.33	-0.50	-0.50	-0.50	-0.27	-0.94	0.83	0.82	0.94	0.94	0.94	0.94	0.94	-0.27
Methyl luteolin deoxyhexosyl hexoside (19)	-0.74	-0.78	-0.78	-0.27	0.33	-0.50	-0.50	-0.50	-0.27	-0.94	0.83	0.82	0.94	0.94	0.94	0.94	0.94	-0.27
Rosmarinic acid (21)	-0.95	-1.00	-1.00	-0.82	-0.33	-0.83	-0.83	-0.83	-0.82	-0.94	0.50	0.27	0.94	0.94	0.94	0.94	0.94	-0.82
Lithospermic acid (24)	-0.74	-0.78	-0.78	-0.27	0.33	-0.50	-0.50	-0.50	-0.27	-0.94	0.83	0.82	0.94	0.94	0.94	0.94	0.94	-0.27
Salvianolic acid H (26)	-0.95	-1.00	-1.00	-0.82	-0.33	-0.83	-0.83	-0.83	-0.82	-0.94	0.50	0.27	0.94	0.94	0.94	0.94	0.94	-0.82
Methyl apigenin deoxyhexosyl hexoside (27)	-0.74	-0.78	-0.78	-0.27	0.33	-0.50	-0.50	-0.50	-0.27	-0.94	0.83	0.82	0.94	0.94	0.94	0.94	0.94	-0.27
Salvianolic acid E/B/L (28)	-0.95	-1.00	-1.00	-0.82	-0.33	-0.83	-0.83	-0.83	-0.82	-0.94	0.50	0.27	0.94	0.94	0.94	0.94	0.94	-0.82
Salvianolic acid E/B/L (29)	-0.74	-0.78	-0.78	-0.27	0.33	-0.50	-0.50	-0.50	-0.27	-0.94	0.83	0.82	0.94	0.94	0.94	0.94	0.94	-0.27
Luteolin (30)	-0.74	-0.78	-0.78	-0.27	0.33	-0.50	-0.50	-0.50	-0.27	-0.94	0.83	0.82	0.94	0.94	0.94	0.94	0.94	-0.27
Salvianolic acid E/B/L (31)	-0.95	-1.00	-1.00	-0.82	-0.33	-0.83	-0.83	-0.83	-0.82	-0.94	0.50	0.27	0.94	0.94	0.94	0.94	0.94	-0.82
α-Thujene (36)	0.95	0.89	0.89	0.54	0.00	0.83	0.83	0.83	0.54	0.94	-0.50	-0.54	-0.94	-0.94	-0.94	-0.94	-0.94	0.54
α-Pinene (37)	0.74	0.89	0.89	0.54	0.00	0.50	0.50	0.50	0.54	0.94	-0.83	-0.54	-0.94	-0.94	-0.94	-0.94	-0.94	0.54
β-Pinene (40)	0.74	0.89	0.89	0.54	0.00	0.50	0.50	0.50	0.54	0.94	-0.83	-0.54	-0.94	-0.94	-0.94	-0.94	-0.94	0.54
Mycene (41)	0.74	0.89	0.89	0.54	0.00	0.50	0.50	0.50	0.54	0.94	-0.83	-0.54	-0.94	-0.94	-0.94	-0.94	-0.94	0.54
α-Terpinene (44)	0.95	0.89	0.89	0.54	0.00	0.83	0.83	0.83	0.54	0.94	-0.50	-0.54	-0.94	-0.94	-0.94	-0.94	-0.94	0.54
p-Cymene (45)	0.74	0.89	0.89	0.54	0.00	0.50	0.50	0.50	0.54	0.94	-0.83	-0.54	-0.94	-0.94	-0.94	-0.94	-0.94	0.54
γ-Terpinene (49)	0.95	0.89	0.89	0.54	0.00	0.83	0.83	0.83	0.54	0.94	-0.50	-0.54	-0.94	-0.94	-0.94	-0.94	-0.94	0.54
Linalool (52)	0.74	0.89	0.89	0.54	0.00	0.50	0.50	0.50	0.54	0.94	-0.83	-0.54	-0.94	-0.94	-0.94	-0.94	-0.94	0.54
Borneol (53)	0.74	0.89	0.89	0.54	0.00	0.50	0.50	0.50	0.54	0.94	-0.83	-0.54	-0.94	-0.94	-0.94	-0.94	-0.94	0.54
Thymol, methyl ether (56)	0.95	0.89	0.89	0.54	0.00	0.83	0.83	0.83	0.54	0.94	-0.50	-0.54	-0.94	-0.94	-0.94	-0.94	-0.94	0.54
Thymol (58)	0.74	0.89	0.89	0.54	0.00	0.50	0.50	0.50	0.54	0.94	-0.83	-0.54	-0.94	-0.94	-0.94	-0.94	-0.94	0.54
Carvacrol (59)	0.95	0.89	0.89	0.54	0.00	0.83	0.83	0.83	0.54	0.94	-0.50	-0.54	-0.94	-0.94	-0.94	-0.94	-0.94	0.54

(*E*)-Caryophyllene (61)	0.95	0.89	0.89	0.54	0.00	0.83	0.83	0.83	0.54	0.94	-0.50	-0.54	-0.94	-0.94	-0.94	-0.94	-0.94	0.54
Caryophyllene oxide (64)	0.74	0.89	0.89	0.54	0.00	0.50	0.50	0.50	0.54	0.94	-0.83	-0.54	-0.94	-0.94	-0.94	-0.94	-0.94	0.54
Phenolic acids	-0.95	-1.00	-1.00	-0.82	-0.33	-0.83	-0.83	-0.83	-0.82	-0.94	0.50	0.27	0.94	0.94	0.94	0.94	0.94	-0.82
Flavonoids	-0.74	-0.78	-0.78	-0.27	0.33	-0.50	-0.50	-0.50	-0.27	-0.94	0.83	0.82	0.94	0.94	0.94	0.94	0.94	-0.27
Monoterpene hydrocarbons	0.95	0.89	0.89	0.54	0.00	0.83	0.83	0.83	0.54	0.94	-0.50	-0.54	-0.94	-0.94	-0.94	-0.94	-0.94	0.54
Oxygeneted monoterpenes	0.74	0.89	0.89	0.54	0.00	0.50	0.50	0.50	0.54	0.94	-0.83	-0.54	-0.94	-0.94	-0.94	-0.94	-0.94	0.54
Sesquiterpene hydrocarbons	0.95	0.89	0.89	0.54	0.00	0.83	0.83	0.83	0.54	0.94	-0.50	-0.54	-0.94	-0.94	-0.94	-0.94	-0.94	0.54
Oxygenated sesquiterpenes	0.74	0.89	0.89	0.54	0.00	0.50	0.50	0.50	0.54	0.94	-0.83	-0.54	-0.94	-0.94	-0.94	-0.94	-0.94	0.54

Table S3. Continued.

Variable	*C. glabrata* MIC	*C. glabrata* MFC	*C. albicans* MIC	*C. albicans* MFC	*C. parapsilosis* MIC	*C. parapsilosis* MFC	VERO CC_{50}	AGS CC_{50}	FaDu CC_{50}	RKO CC_{50}	Anti-HHV1 CPE	Anti-Ad5 CPE	Anti-HHV1 titer	Anti-Ad5 titer	Anti-HHV1 load
Apigenin 6,8-di-*C*-glucoside (8)	0.89	0.94	0.94	0.89	0.95	0.89	0.74	0.95	0.95	0.95	-0.78	-0.78	-0.78	-0.78	-0.78
Hydroxy kaempferol hexuronide (10)	0.89	0.94	0.94	0.89	0.95	0.89	0.74	0.95	0.95	0.95	-0.78	-0.78	-0.78	-0.78	-0.78
Luteolin 7-*O*-rutinoside (11)	0.89	0.94	0.94	0.89	0.74	0.89	0.95	0.74	0.74	0.74	-1.00	-1.00	-1.00	-1.00	-1.00
Luteolin 7-*O*-glucuronide (15)	0.89	0.94	0.94	0.89	0.95	0.89	0.74	0.95	0.95	0.95	-0.78	-0.78	-0.78	-0.78	-0.78
Methyl luteolin deoxyhexosyl hexoside (19)	0.89	0.94	0.94	0.89	0.95	0.89	0.74	0.95	0.95	0.95	-0.78	-0.78	-0.78	-0.78	-0.78
Rosmarinic acid (21)	0.89	0.94	0.94	0.89	0.74	0.89	0.95	0.74	0.74	0.74	-1.00	-1.00	-1.00	-1.00	-1.00
Lithospermic acid (24)	0.89	0.94	0.94	0.89	0.95	0.89	0.74	0.95	0.95	0.95	-0.78	-0.78	-0.78	-0.78	-0.78
Salvianolic acid H (26)	0.89	0.94	0.94	0.89	0.74	0.89	0.95	0.74	0.74	0.74	-1.00	-1.00	-1.00	-1.00	-1.00
Methyl apigenin deoxyhexosyl hexoside (27)	0.89	0.94	0.94	0.89	0.95	0.89	0.74	0.95	0.95	0.95	-0.78	-0.78	-0.78	-0.78	-0.78
Salvianolic acid E/B/L (28)	0.89	0.94	0.94	0.89	0.74	0.89	0.95	0.74	0.74	0.74	-1.00	-1.00	-1.00	-1.00	-1.00
Salvianolic acid E/B/L (29)	0.89	0.94	0.94	0.89	0.95	0.89	0.74	0.95	0.95	0.95	-0.78	-0.78	-0.78	-0.78	-0.78
Luteolin (30)	0.89	0.94	0.94	0.89	0.95	0.89	0.74	0.95	0.95	0.95	-0.78	-0.78	-0.78	-0.78	-0.78
Salvianolic acid E/B/L (31)	0.89	0.94	0.94	0.89	0.74	0.89	0.95	0.74	0.74	0.74	-1.00	-1.00	-1.00	-1.00	-1.00
α-Thujene (36)	-0.78	-0.94	-0.94	-0.78	-0.74	-0.78	-0.95	-0.74	-0.74	-0.74	0.89	0.89	0.89	0.89	0.89
α-Pinene (37)	-1.00	-0.94	-0.94	-1.00	-0.95	-1.00	-0.74	-0.95	-0.95	-0.95	0.89	0.89	0.89	0.89	0.89
β-Pinene (40)	-1.00	-0.94	-0.94	-1.00	-0.95	-1.00	-0.74	-0.95	-0.95	-0.95	0.89	0.89	0.89	0.89	0.89

Mycene (41)	-1.00	-0.94	-0.94	-1.00	-0.95	-1.00	-0.74	-0.95	-0.95	-0.95	0.89	0.89	0.89	0.89	0.89
α-Terpinene (44)	-0.78	-0.94	-0.94	-0.78	-0.74	-0.78	-0.95	-0.74	-0.74	-0.74	0.89	0.89	0.89	0.89	0.89
p-Cymene (45)	-1.00	-0.94	-0.94	-1.00	-0.95	-1.00	-0.74	-0.95	-0.95	-0.95	0.89	0.89	0.89	0.89	0.89
γ-Terpinene (49)	-0.78	-0.94	-0.94	-0.78	-0.74	-0.78	-0.95	-0.74	-0.74	-0.74	0.89	0.89	0.89	0.89	0.89
Linalool (52)	-1.00	-0.94	-0.94	-1.00	-0.95	-1.00	-0.74	-0.95	-0.95	-0.95	0.89	0.89	0.89	0.89	0.89
Borneol (53)	-1.00	-0.94	-0.94	-1.00	-0.95	-1.00	-0.74	-0.95	-0.95	-0.95	0.89	0.89	0.89	0.89	0.89
Thymol, methyl ether (56)	-0.78	-0.94	-0.94	-0.78	-0.74	-0.78	-0.95	-0.74	-0.74	-0.74	0.89	0.89	0.89	0.89	0.89
Thymol (58)	-1.00	-0.94	-0.94	-1.00	-0.95	-1.00	-0.74	-0.95	-0.95	-0.95	0.89	0.89	0.89	0.89	0.89
Carvacrol (59)	-0.78	-0.94	-0.94	-0.78	-0.74	-0.78	-0.95	-0.74	-0.74	-0.74	0.89	0.89	0.89	0.89	0.89
(*E*)-Caryophyllene (61)	-0.78	-0.94	-0.94	-0.78	-0.74	-0.78	-0.95	-0.74	-0.74	-0.74	0.89	0.89	0.89	0.89	0.89
Caryophyllene oxide (64)	-1.00	-0.94	-0.94	-1.00	-0.95	-1.00	-0.74	-0.95	-0.95	-0.95	0.89	0.89	0.89	0.89	0.89
Phenolic acids	0.89	0.94	0.94	0.89	0.74	0.89	0.95	0.74	0.74	0.74	-1.00	-1.00	-1.00	-1.00	-1.00
Flavonoids	0.89	0.94	0.94	0.89	0.95	0.89	0.74	0.95	0.95	0.95	-0.78	-0.78	-0.78	-0.78	-0.78
Monoterpene hydrocarbons	-0.78	-0.94	-0.94	-0.78	-0.74	-0.78	-0.95	-0.74	-0.74	-0.74	0.89	0.89	0.89	0.89	0.89
Oxygeneted monoterpenes	-1.00	-0.94	-0.94	-1.00	-0.95	-1.00	-0.74	-0.95	-0.95	-0.95	0.89	0.89	0.89	0.89	0.89
Sesquiterpene hydrocarbons	-0.78	-0.94	-0.94	-0.78	-0.74	-0.78	-0.95	-0.74	-0.74	-0.74	0.89	0.89	0.89	0.89	0.89
Oxygenated sesquiterpenes	-1.00	-0.94	-0.94	-1.00	-0.95	-1.00	-0.74	-0.95	-0.95	-0.95	0.89	0.89	0.89	0.89	0.89

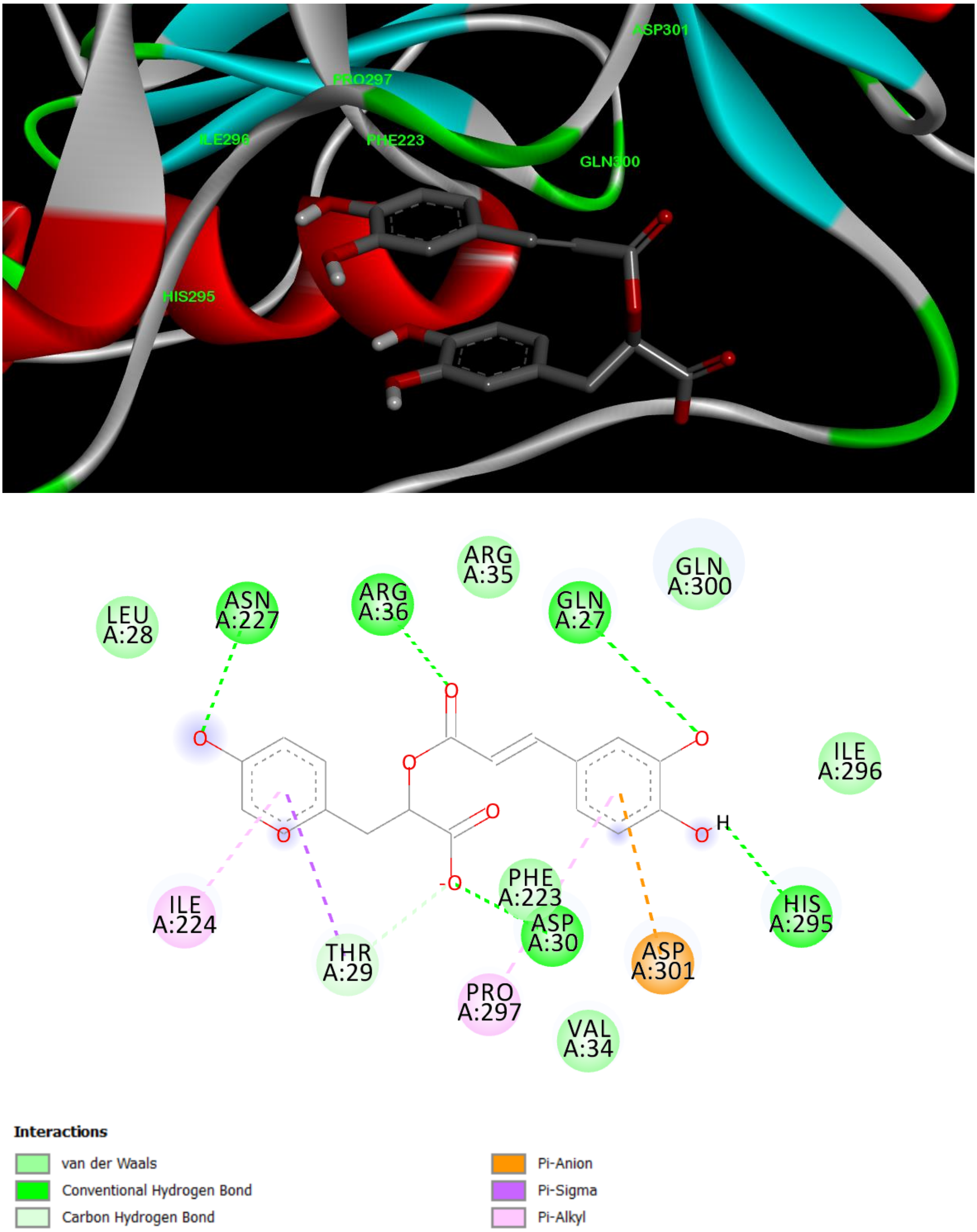

Figure S5. Binding of rosmarinic acid to gD (PDB ID: 2C36): 3D representation with labeled amino acid residues suspected to be involved in binding to nectin-1 (top); Visualization of ligand-protein interactions using Discovery Studio Visualizer 2019 (bottom).

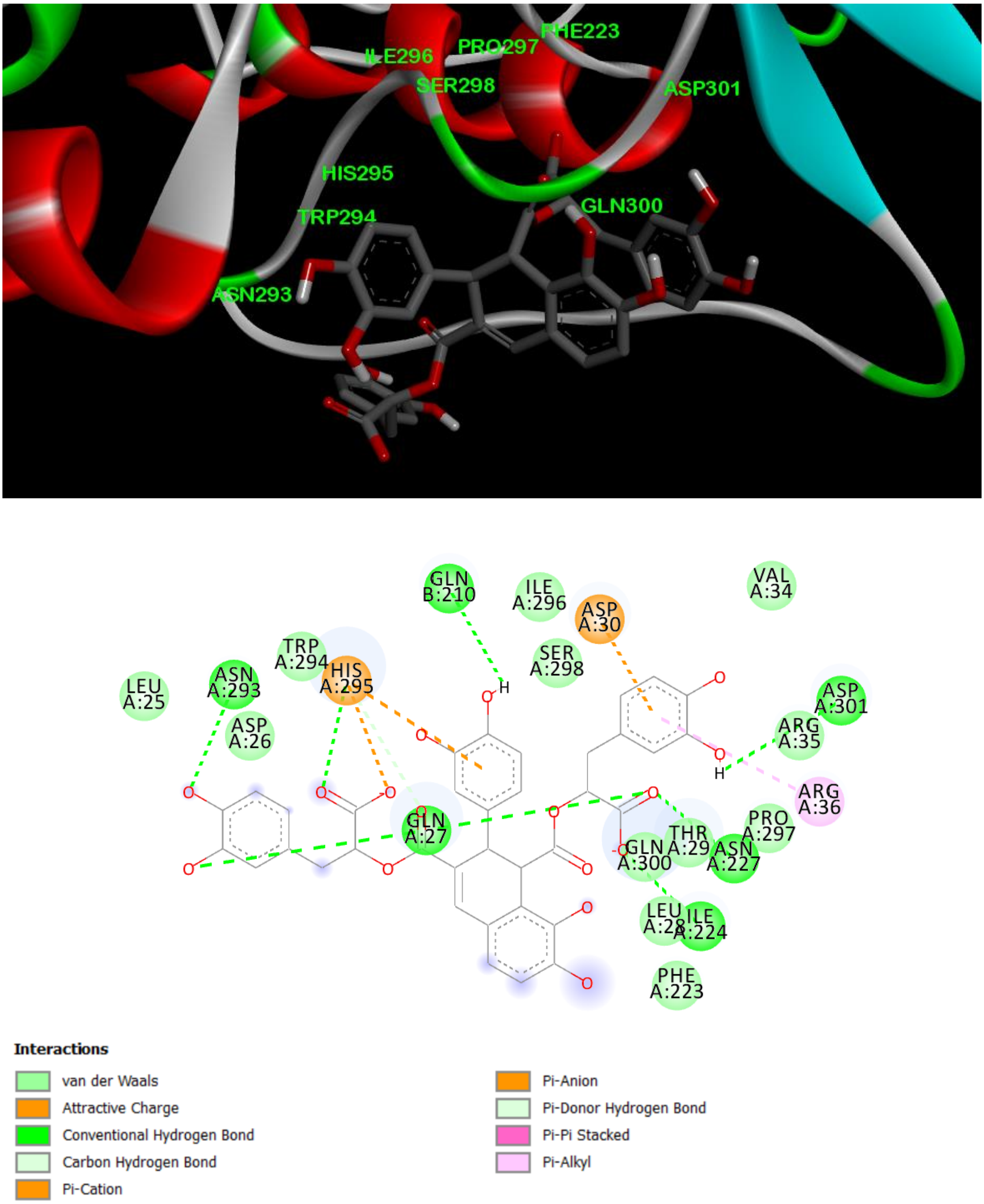

Figure S6. Binding of salvianolic acid L to gD (PDB ID: 2C36): 3D representation with labeled amino acid residues suspected to be involved in binding to nectin-1 (top); Visualization of ligand-protein interactions using Discovery Studio Visualizer 2019 (bottom).

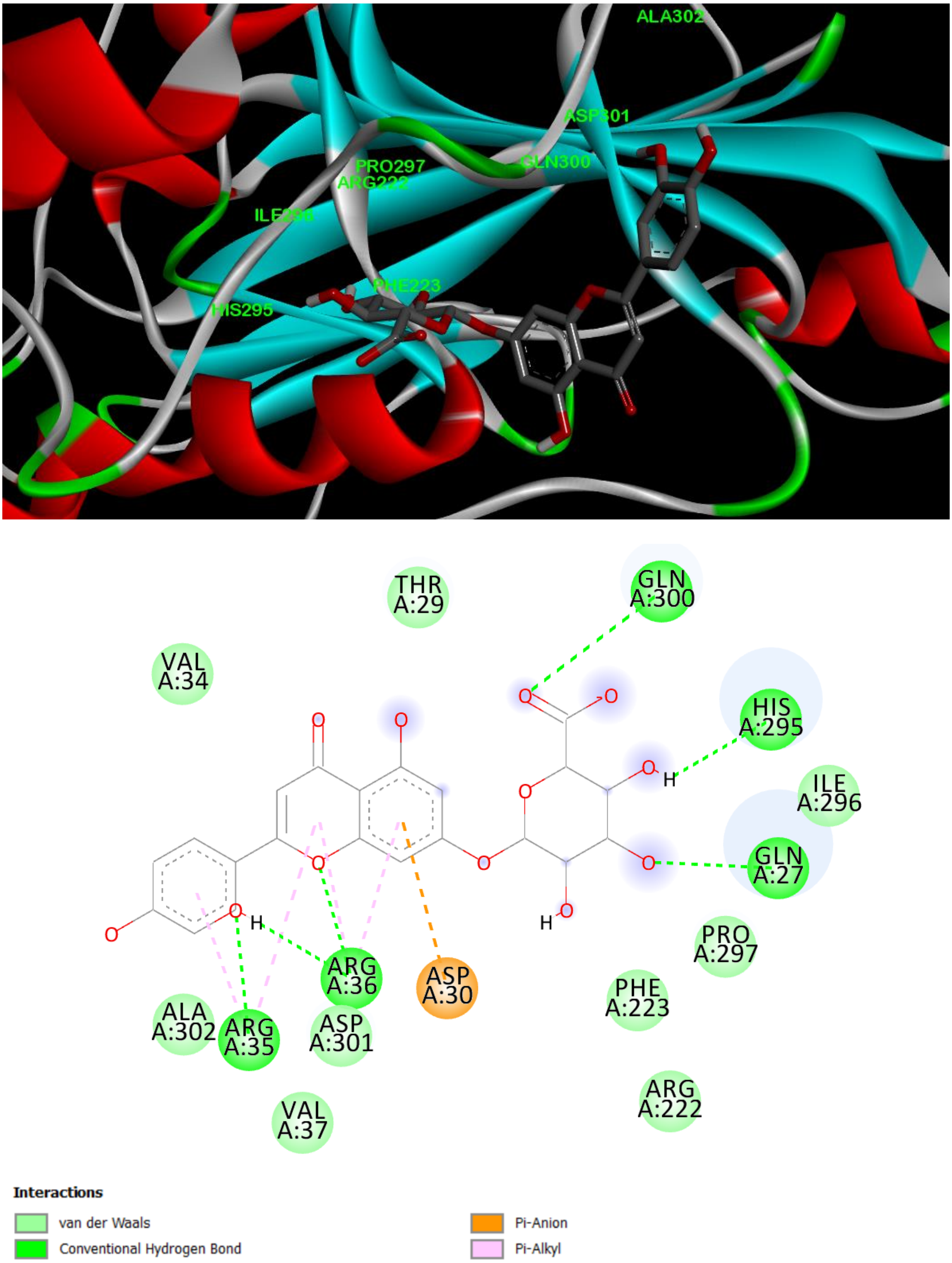

Figure S7. Binding of luteolin 7-*O*-glucuronide to gD (PDB ID: 2C36): 3D representation with labeled amino acid residues suspected to be involved in binding to nectin-1 (top); Visualization of ligand-protein interactions using Discovery Studio Visualizer 2019 (bottom).
